# Restoring degraded agricultural peatlands: how rewetting, biochar, and iron sulphate synergistically modify microbial hotspots and carbon storage

**DOI:** 10.1007/s42773-025-00501-y

**Published:** 2025-09-10

**Authors:** Peduruhewa H. Jeewani, Robert W. Brown, Jennifer M. Rhymes, Chris D. Evans, Dave R. Chadwick, Davey L. Jones

**Affiliations:** 1 School of Environmental and Natural Sciences, Bangor University, Bangor, Gwynedd LL57 2UW UK; 2 UK Centre for Ecology and Hydrology, Bangor, Gwynedd LL57 2UW UK

**Keywords:** Soil solution, Hydrolytic enzymes, Sustainable agriculture, Soil microbes, Fe gate, Histosol

## Abstract

The draining and conversion of peatlands for agriculture has led to their degradation globally, diminishing their carbon (C) storage capacity and functioning. However, rewetting, alongside the addition of organic/inorganic amendments, has the potential to accelerate peat formation and C accrual. The aim of this experiment was therefore to examine the combined benefits of altering water table depth and adding organic (e.g., biochar, paper waste, biosolids, cereal straw; 20 t C ha^−1^) and inorganic (e.g., FeSO_4_; 0.5 t ha^−1^) materials on net C storage and peatland functioning (i.e., microbial communities, greenhouse gas emissions and biogeochemical cycling). The experiment consisted of outdoor agricultural peat mesocosms monitored over 1 year. The relative effectiveness of the amendments in preserving peat-C (t C ha^−1^) followed the series: *Miscanthus* biochar (18.9 t C ha^−1^) > *Miscanthus* residues (17.3 t C ha^−1^) > biosolids (17.2 t C ha^−1^) > cereal straw (14.5 t C ha^−1^) > paper waste (13.3 t C ha^−1^) based on C additional rate (20 t C ha^−1^). Overall, a high-water table combined with biochar and FeSO_4_ addition was the most effective at suppressing enzyme activity (e.g., β-glucosidase, phenol oxidase, cellobiase), methanogen activity (e.g., *Methanosarcina*) and peat mineralization rate. We ascribe this in part to changes in the fungal and bacterial community structure (e.g., reductions in Actinobacteria by − 22% and Ascomycota by − 61%). FeSO_4_ also increased the Fe-bound C content in the non-rewetted treatment, supporting the ‘iron gate’ mechanism for C preservation. The mechanisms behind our results appear to be both abiotic (affecting SOC solubility through changes in redox conditions and Fe–C interactions) and biotic (via shifts in microbial community and enzyme activities), creating conditions that enhance C preservation. These findings provide evidence for implementing biochar and FeSO_4_ amendments alongside water table management as practical, scalable strategies for restoring C storage capacity in agricultural peatlands.

**Supplementary Information:**

The online version contains supplementary material available at 10.1007/s42773-025-00501-y.

## Highlights

- Rewetting with biochar and FeSO_4_ suppresses decomposers and enhances peat C storage.
- FeSO_4_ suppresses methanogens and increases Fe-protected C in aerobic conditions.
- Biochar modifies the microbial community and reduces hydrolytic enzyme activity.
- Strategic amendment combinations reduce GHG emissions while preserving peat soil C.
- Raising water tables prevents dominance of Actinobacteria and Ascomycota decomposers.

**Supplementary Information:**

The online version contains supplementary material available at 10.1007/s42773-025-00501-y.

## Introduction

Peatlands account for approximately 2.84% of the global land area, constituting the largest terrestrial carbon (C) reservoir (Evans et al. 2017; Evans et al. 2023). Despite their critical role in carbon storage, an estimated 20% of the world’s peatlands are currently under agricultural use, which significantly alters their natural carbon dynamics and contributes to greenhouse gas emissions (Bonn et al. 2016; Xu et al. 2018; Kwon et al. 2022). Drainage of lowland peat soils is a common method of increasing agricultural productivity, leading to significant degradation and loss of C through mineralization (Andersen et al. 2013; Brouns et al. 2016). Rewetting of peat soils and application of soil amendments (for example, biochar; the product of pyrolysis of organic feedstock) are common restoration practices (Evans et al. 2023; Deshoux et al. 2023; Jeewani et al. 2025), yet our understanding of the belowground biogeochemistry, particularly C and nitrogen (N) cycling, is still incomplete. Thus, it is important to consider how peat soil biogeochemistry is affected by these new practices, particularly when estimating the current and future responses of peatland C balance.

Peatlands contain a diverse fungal community with significant metabolic diversity that play a key role in organic matter transformation (Andersen et al. 2013). Among these fungi, Ascomycota is believed to be the most abundant phylum responsible for aerobic decomposition in peatlands (Pankratov et al. 2006). Peltoniemi et al. (2009) observed that the peat fungal community varied between sites representing the prevailing nutrient and water table regime. However, contrasting results have been observed among the peat fungal community in response to lowering of the water table (Peltoniemi et al. 2009; Wang et al. 2024b). Changes in the composition and size of the bacterial community have also been observed in response to changes in water table level (Deshoux et al. 2023). Typically, the community becomes less diverse when the water table is raised, a change which has been ascribed to the imposition of anaerobic conditions, decreased competition and increased rates of predation (Andersen et al. 2013). In peatlands, the boundary zone between the oxic and anoxic layers, where CH_4_ and O_2_ are both present, also creates an ideal niche for methanotrophs, the most prevalent bacterial phyla of which are *Proteobacteria* (*gamma*- and *alpha*-*Proteobacteria*) and *Verrucomicrobia* (Op den Camp et al. 2009). Given the diverse microbial responses to environmental conditions in peatlands, it is essential to investigate how microbial community structure and function change in response to management interventions such as rewetting and amendment addition, particularly regarding their effects on C storage and greenhouse gas (GHG) emissions.

Incorporation or surface broadcasting of organic amendments re-introduces C that may have previously been lost due to peatland drainage and cultivation. However, the success in restoring C is likely regulated by the biogeochemistry of the added substrate, moisture status and their combined impact on the composition and functioning of the soil microbial community (Eilers et al. 2010; Butterbach-Bahl et al. 2013; Kandel et al. 2019; Brown et al. 2022). For example, due to its aromatic structure, biochar-derived C is highly persistent in soil, while other C substrates (e.g., straw) may be less persistent and generate unwanted CH_4_ emissions (Liu et al. 2014; Wang et al. 2024b). To date, however, our mechanistic understanding of the fate and behavior of different C substrates in rewetted agricultural peatlands remains poor.

Peat soil biogeochemistry can be strongly influenced by the presence of alternative electron acceptors (e.g., Fe-oxyhydroxides, NO_3_^−^, SO_4_^2−^), suggesting that they have the potential to be used to manipulate microbial functioning during peatland restoration (Dean et al. 2018; Richy et al. 2024). For example, incubation experiments have observed that the addition of Fe^3+^ and SO_4_^2−^ may limit both denitrification and methanogenesis (Wen et al. 2019; Jeewani et al. 2025). The presence of SO_4_^2−^ may also promote the growth of SO_4_^2−^-reducing bacteria such as *Desulfovibrio spp., Desulfosarcina spp.* and *Desulfobacter spp* and archaea (*Archaeoglobus spp.*), leading to increased competition and a suppression of methanogen activity (Pester et al. 2012; Ozuolmez et al. 2015). Furthermore, earlier field studies have demonstrated that rewetting iron-rich peat soils can still lead to substantial CH₄ emissions, even though methanogenesis is expected to be suppressed by the presence of iron oxides (Yamada et al. 2014). However, the impact of FeSO_4_ addition on the GHG release and net C storage in agricultural peat soils remains poorly understood.

To the best of our knowledge, this is the first study to investigate how the combined application of inorganic and organic amendments, along with water table adjustments, influences microbial community dynamics in agricultural peatland soils. In this study, our goal was to identify the most effective amendments for enhancing soil carbon content without promoting the decomposition of soil organic matter (SOM) or increasing net GHG emissions. We hypothesized that: 1) Under conditions of elevated water table, the addition of high C:N ratio amendments (e.g., *Miscanthus* biochar) will limit nitrogen availability to decomposers, thereby constraining microbial growth and enzyme-mediated decomposition processes via nutrient immobilization, relative to low C:N treatments; 2) Low C:N organic amendments (e.g., biosolids, paper waste) will stimulate rapid microbial turnover and SOM decomposition by promoting the growth of copiotrophs (r-strategists), leading to increased enzyme activity and CO₂ fluxes; 3) FeSO₄ addition will suppress methanogenesis by favoring alternative terminal electron-accepting processes (e.g., iron reduction), leading to shifts in the abundance of methanogenic archaea and lower CH₄ emissions.

## Materials and methods

### Site information

Intact soil cores were obtained from a commercially managed lowland agricultural peat field in Nottinghamshire, UK (53°27’N, 00°54'W) in March 2022. The site is a level, drained lowland fen characterized by an approximately 0.5-m-thick organic layer resting atop mineral soil and had been under intensive agricultural production for over 100 years. The average annual temperature of the site is 10.3 °C and annual rainfall of 1162 mm, and the soil is classified as an Ombric Sapric Histosols (Mantel, et al., 2023)). Intact soil cores (*n* = 56; depth = 50 cm) were collected using PVC pipes (⌀ = 20 cm, height = 60 cm). The intact soil cores were transported to Bangor University, where they were kept outdoors throughout the entire 365-day measurement period.

### Basic properties of organic amendments and soil

Initial soil (0–10 cm depth) and organic amendment characteristics were quantified at the start of the experiment. The pH and electrical conductivity (EC) of soil samples were tested in 1:2.5 (w/v) soil-to-distilled water suspensions using a standard probe. Bulk density was measured using the fixed-volume ring technique, following the procedure detailed by Blake and Hartge (1986). Total carbon (TC) and total nitrogen (TN) levels in both soil samples and organic amendments were analyzed using oven-dried (80 °C for 24 h), finely ground material on a TruSpec® CN Analyzer (Leco Corp., St. Joseph, MI). SOM content was assessed through the loss-on-ignition method, conducted in a muffle Furnace at 450 °C for a duration of 16 h. Dissolved organic carbon (DOC) and total dissolved nitrogen (TDN) were quantified from a 0.5 M potassium sulfate (K₂SO₄) extraction using a Multi N/C 2100/2100 analyzer (Analytik Jena AG, Jena, Germany). Nutrient concentrations including ammonium (NH₄⁺), nitrate (NO₃⁻), phosphate (PO₄^3^⁻), and sulfate (SO₄^2^⁻) in the amendments were extracted with distilled water at a 1:5 (w/v) ratio and analyzed spectrophotometrically using a Power Wave-Xs microplate reader (BioTek Instruments Inc., Winooski, VT, USA), based on established colorimetric protocols (Murphy & Riley, 1962; Bradfield & Cooke, 1985; Kuo & Sparks, 1996). Microbial biomass carbon (MBC) was estimated through the chloroform fumigation–extraction technique, as described by Brookes et al. (1985) and Vance et al. (1987). The biochar material was characterized by determining its atomic hydrogen-to-carbon (H/C) ratio, and the stable polyaromatic carbon (SPAC) content was measured via hydropyrolysis (Hypy) following the methodology of Ascough et al. (2009). The detailed characteristics of the soil and organic amendments are provided in Table S1 (Jeewani et al. 2025).

### Experimental setup

The mesocosm experiment comprised 14 treatments (each with four replicates), incorporating five different organic amendments that varied in their C:N ratios. The treatments were: (i) Control soil cores with a water table (WT) at − 40 cm (Control + LW; to represent no change of management), (ii) Control soil cores with a saturated water table at the soil surface (0 cm; Control + HW), (iii) high WT + pyrolysed *Miscanthus giganteus* wood chip (biochar; pyrolysed at 450 °C, 30 min; C:N ratio = 258; Biochar + HW), (iv) high WT + commercial paper waste (C:N ratio = 155; Paperwaste + HW), (v) high WT + *M. giganteus-*derived chip (C:N ratio = 96; M.chip + HW), (vi) high WT + Cereal straw (*Hordeum vulgare* L.) (C:N ratio = 63; C.straw + HW), and (vii) high WT + anaerobically digested biosolids sourced from a high-capacity urban wastewater treatment plant (C:N ratio = 10; Biosolids + HW). Each treatment was assessed with or without the addition of FeSO_4_ (Table S2; Fig.S3). All organic substrates were applied at a rate of 20 t C ha⁻^1^ to simulate field-level organic residue management practices (Jones et al. 2012; Pandit et al. 2018). while FeSO₄ was added at in accordance with Wen et al. (2019). The FeSO₄·7H₂O addition rate (0.5 t ha⁻^1^) was selected based on prior peatland amendment studies aiming to stimulate Fe–C complexation and suppress methanogenesis without exceeding levels known to cause toxicity or alter redox buffering capacity (Wen et al. 2019; Hu et al. 2020). Both inorganic (FeSO₄) and organic amendments were manually incorporated into the upper 10 cm of the soil to emulate mechanical harrowing under field conditions.

Each mesocosm was positioned within a larger external container filled with water, functioning as a bund system. Drainage holes were installed to regulate the water table at either a high level (0 cm) or a low level (–40 cm). Prior to initiating the experiment, mesocosms were stabilized at their designated water table depths for a period of three days. Throughout the experimental period, water levels were sustained via natural rainfall, supplemented with tap water during dry intervals. The selected water table settings were intended to establish primarily anaerobic conditions in the high-water table (HW) or 're-wetted' treatments, and predominantly aerobic conditions in the low water table (LW) control, simulating conventional drainage-based agricultural practices.

### Soil solution chemistry

Horizontally installed rhizon samplers (Rhizosphere Research Products, Wageningen, The Netherlands) were used to collect soil solution from soil depths of 5 cm (in the treatment layer) and 25 cm (below the treatment layer). Samples were collected on days 1, 3, 5 and 7 then weekly and monthly, parallel to the GHG sample collections (Jeewani et al. 2025). On collection, the samples were stored at (−20 °C for < 14 days) until analysis for TDN, DOC, NH_4_^+^, NO_3_^−^, PO_4_^3−^ and SO_4_^2−^, and were quantified using the methods described in Sect. 2.2. At each sampling event, in situ measurements of soil pH, temperature (T), redox potential (Eh), and electrical conductivity (EC) were conducted within the upper 5 cm of the soil profile using a Sension + MM150 portable sensor system (Hach UK, Manchester, UK). Additionally, the DOM (filtered through 0.45 µm) was measured by specific UV absorbance at 254 nm using a Cary 60 UV–vis spectrophotometer (UV–Vis, Agilent Technologies, UK). This has been shown to be indicative of the hydrophobic organic acid fraction, as well as aromatic content and molecular weight of compounds present (Spencer et al. 2012; Chowdhury 2013).

### Fe-bound organic C determination

Fe-organic carbon complexes were quantified using the dithionite citrate bicarbonate (DCB) method after 180 days, following Wang et al. (2017). Briefly, lyophilized soil (0–10 cm depth) was mixed with a buffer solution at a1:60 (w/v) ratio: a solution containing 0.27 M trisodium citrate and 0.11 M sodium bicarbonate, adjusted to pH 7.3 and heated to 80 °C. Then, 0.5 g of sodium dithionite was added as a reducing agent, and the mixture was maintained at 60–80 °C for 15 min. For the control treatment, the soil samples were extracted with NaCl at an equivalent ionic strength instead of undergoing DCB extraction. The residues were subsequently rinsed three times with 1 M NaCl, then were oven-dried for SOC analysis, following the procedure outlined previously. The concentration of Fe-bound organic carbon was determined by calculating the difference in SOC content between the control and the reduction treatments. Soil Fe(II) and Fe(III) contents were analyzed using the ferrozine-ultraviolet absorbance method (Wang et al. 2017). Specifically, fresh soil (0–10 cm depth) was extracted with 0.5 M HCl overnight (1:25 w/v). Fe(II) concentration was quantified by measuring absorbance at 562 nm with a UV–Vis spectrophotometer, following reaction with a 5 mM ferrozine solution (Stookey, 1970). Total iron was quantified after reduction with 2% hydroxylamine hydrochloride, with Fe(II) subsequently measured as described above. The amount of Fe(III) was calculated by subtracting the Fe(II) value from the total iron and expressed as mg g⁻^1^ soil.

### Extracellular enzyme activity

Enzyme activity kinetics were evaluated using fluorogenic 4-methylumbelliferone (MUF) substrates (Razavi et al. 2015). The MUF substrates were diluted with sterile MES buffer to the required concentrations, and enzyme activities were measured across a gradient of substrate concentrations (0, 10, 20, 30, 40, 50, 100, 200 μmol g⁻^1^ soil). Briefly, soil suspensions (1:50, w/v; soil-to-deionized H₂O) were shaken for 2 min (Koch et al. 2007). Subsequently, 50 μL of the soil suspension was combined with 50 μL of buffer (pH 6.5) and 100 μL of the appropriate substrate solution in a 96-well microplate (Puregrade, Germany). Calibration solutions were prepared by combining 50 μL of the soil suspension with varying concentrations of MUF to produce a range from 0 to 1.2 mM (Ali et al. 2015). Fluorescence was determined (λEX = 355 nm, λEM = 460 nm) using a Victor3 1420-050 multi-label counter (Perkin Elmer, USA). Enzyme activity was recorded at 25 °C after 0.5, 1, and 2 h (Razavi et al. 2015).

Phenol oxidase activity was analysed following the method described by Saiya-Cork et al. (2002). In brief, fresh soil (0.2 g at 0–10 cm depth) was mixed with 125 mL of 50 mM Tris buffer (pH 7.8) for 2.5 min using a magnetic stirrer. From the resulting suspension, 200 µL was transferred into 96-well microplates, with eight replicate wells per sample for each assay. Subsequently, 50 µL of a 5 mM L-3,4-dihydroxyphenylalanine (L-DOPA) solution was added to each well. The plates were incubated in the dark at 20 °C for 4 h. Absorbance was then measured at 450 nm using a Multi-Mode Microplate Reader (Synergy Mx, BioTek Instruments Inc., USA). Enzyme activity was reported as mmol h⁻^1^ g⁻^1^ dry soil (Razavi et al. 2015).

### DNA sequencing

Soil DNA was extracted from samples collected at the 0–10 cm layer at the conclusion of the 365-day experiment, using the Zymo Research Soil DNA Kit (Zymo Research, USA). The quantity of extracted DNA was first evaluated through 1% agarose gel electrophoresis, followed by assessment of DNA concentration and purity using a NanoDrop spectrophotometer (Thermo Scientific). Polymerase chain reaction (PCR) amplifications were employed using an ABI 9700 thermocycler (Thermo Fisher Scientific, Waltham, MA, USA). For bacterial community analysis, the V3-V4 region of the 16S rRNA gene (~ 450–550 bp) was targeted using the primers 341F (CCTAYGGGRBGCASCAG) and 806R (GGACTACNNGGGTATCTAAT). Fungal community composition was assessed by amplifying the ITS2 region (~ 380 bp) with primers ITS3 (GCATCGATGAAGAACGCAGC) and ITS4 (TCCTCCGCTTATTGATATGC).

PCR reactions were prepared in a total volume of 25 μL, consisting of 12.5 μL of 2 × Taq Plus Master Mix, 3 μL of BSA (2 ng μL⁻^1^), 1 μL of each forward and reverse primer (5 μM), 2 μL of template DNA, and 5.5 μL of nuclease-free water. The thermal cycling protocol included an initial denaturation at 95 °C for 5 min, followed by 28 cycles of denaturation at 95 °C for 45 s, annealing at 55 °C for 50 s, and extension at 72 °C for 45 s. A final extension was performed at 72 °C for 10 min, with a subsequent cooling step at 4 °C. Amplified products were confirmed through 1% agarose gel electrophoresis and subsequently purified using the Agencourt AMPure XP kit. Paired-end sequencing was performed using the Illumina MiSeq PE300 platform for high-throughput analysis. All PCR amplification and sequencing for bacterial and fungal communities were carried out by Novogene Co., Ltd. (Beijing, China).

### Statistical analyses

Statistical analyses of soil variables across treatments were conducted using both one-way and two-way ANOVA in SPSS version 24 (IBM Corp., Chicago, IL, USA). Before analysis, data were tested for normality and homogeneity of variance using the Shapiro–Wilk and Levene's tests, respectively. When assumptions were met, treatment differences were further examined using Tukey’s post-hoc test. For datasets that did not meet these assumptions, log₁₀ or square root transformations were applied as appropriate. Replication (n = 4) was selected based on constraints typical of mesocosm experiments. A post hoc power analysis indicated that the design had sufficient power (≥ 80%) to detect effect sizes of d ≥ 1.2 at α = 0.05. All statistical comparisons were made within individual sampling dates. IBM SPSS version 29 was used to perform ANOVA tests (significance level: *p* < 0.05).

Visualization of temporal trends in soil solution chemistry and other biophysicochemical properties was performed using Origin 2022 (Origin Lab Corp., USA). Microbial community analyses were conducted in R (version 4.4.2; R Core Team, 2024). Microbial community composition differences were assessed using Principal Coordinates Analysis (PCoA) based on Bray–Curtis dissimilarity matrices through the ‘vegan’ package (Oksanen 2013). Heatmaps representing soil physicochemical parameters and microbial community structure were generated using the ‘pheatmap’ package to explore relationships between environmental variables and microbial variation.

To assess the influence of specific environmental factors (e.g., pH, NH₄⁺, TN, DOC, total Fe, redox potential, phenol oxidase, β-glucosidase, cellobiase activity, and temperature) on microbial communities, distance-based linear modeling (distLM) was performed using the distLM_forward3 software (Anderson 2003).

Microbial co-occurrence networks were constructed to explore interactions within the soil microbiome. The 300 most abundant bacterial amplicon sequence variants (ASVs) and 200 most abundant Fungal ASVs were selected to minimize spurious correlations while ensuring comparability across treatments. These ASVs represented roughly 54% and 80% of the total bacterial and fungal abundances, respectively. To construct robust microbial co-occurrence networks, pairwise associations between taxa were first calculated using Spearman rank correlation, both of which are suitable for compositional microbial abundance data. To ensure biological relevance and reduce noise, weak or statistically insignificant associations were excluded by applying a correlation threshold (|r|< 0.6) and a significance level (*p* > 0.05). The significance of each correlation was further assessed through bootstrap resampling, allowing us to evaluate whether observed interactions deviated from those expected by chance. To account for the large number of comparisons and minimize the risk of false positives, multiple testing correction was performed using the Benjamini–Hochberg false discovery rate (FDR) method. Only statistically significant and strong correlations that passed these criteria were retained for network construction and subsequent topological analysis. Network construction and visualization were performed using Cytoscape version 3.5.1 (Shannon et al. 2003) and Gephi (https://gephi.github.io/).

## Results

### Physicochemical characteristics of soil and pore water

**Fig. 1:**
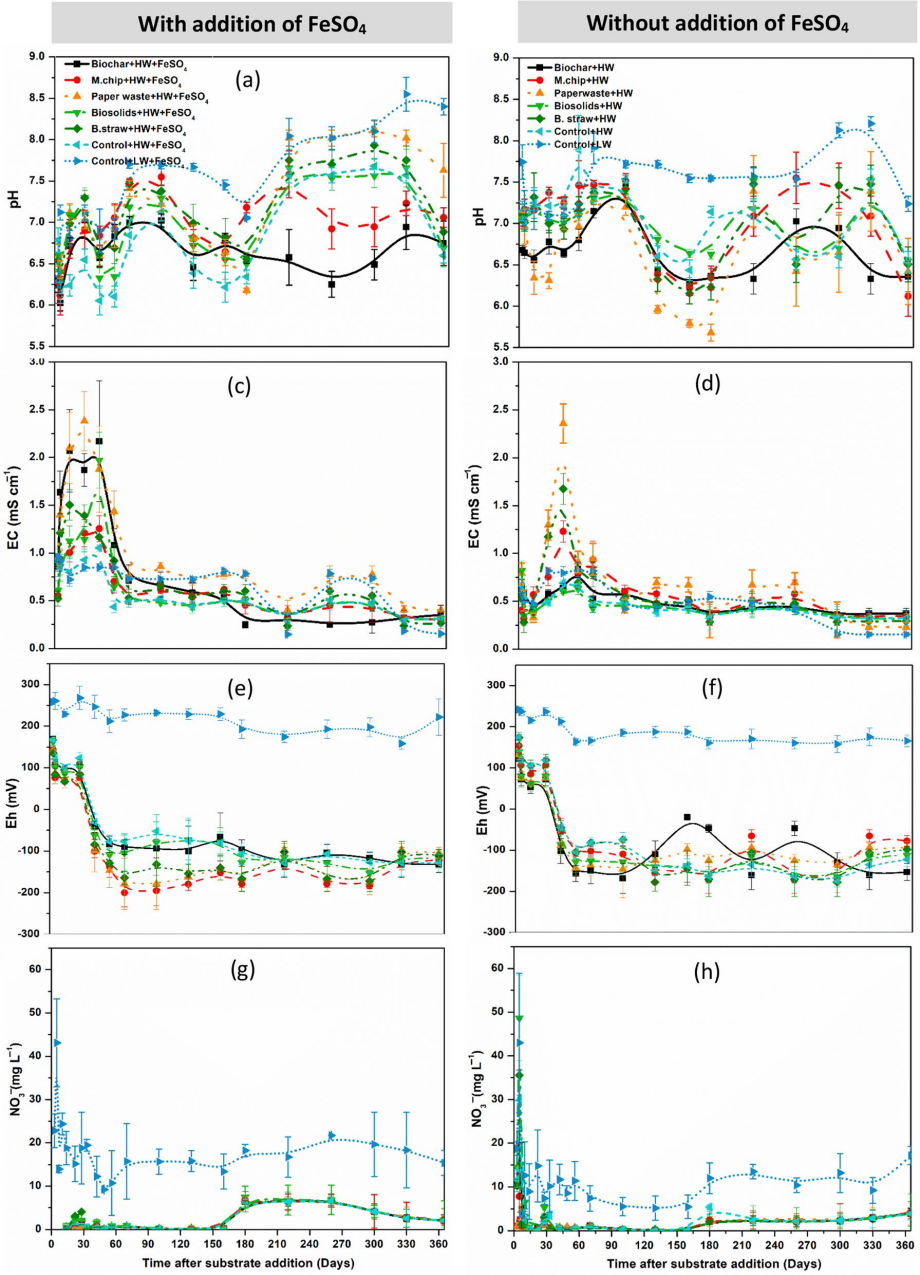
Effect of water table manipulation, organic C amendment loaded at 20 t C ha^−1^ and FeSO_4_ addition on changes in soil chemical properties in a lowland agricultural peat soil over a one-year in an outdoor mesocosm experiment. Soil pH (**a** and **b**), soil EC (**c** and **d**), soil redox potential (**e** and **f**), nitrate concentration (**g** and **h**). The C amendments were loaded at 20 t C ha^−1^ and included biochar (pyrolysed *Miscanthus giganteus* wood chip), commercial paper waste, *M. giganteus* derived chip, barley straw and advanced anaerobically digested biosolids. Water table heights were low water table (LW; − 40 cm, representing no change of management), high water table (HW; 0 cm, representing rewetting) and FeSO_4_ addition ± at a rate of 0.5 t ha^−1^. Values represent mean ± standard error (*n* = 4)

**Fig. 2:**
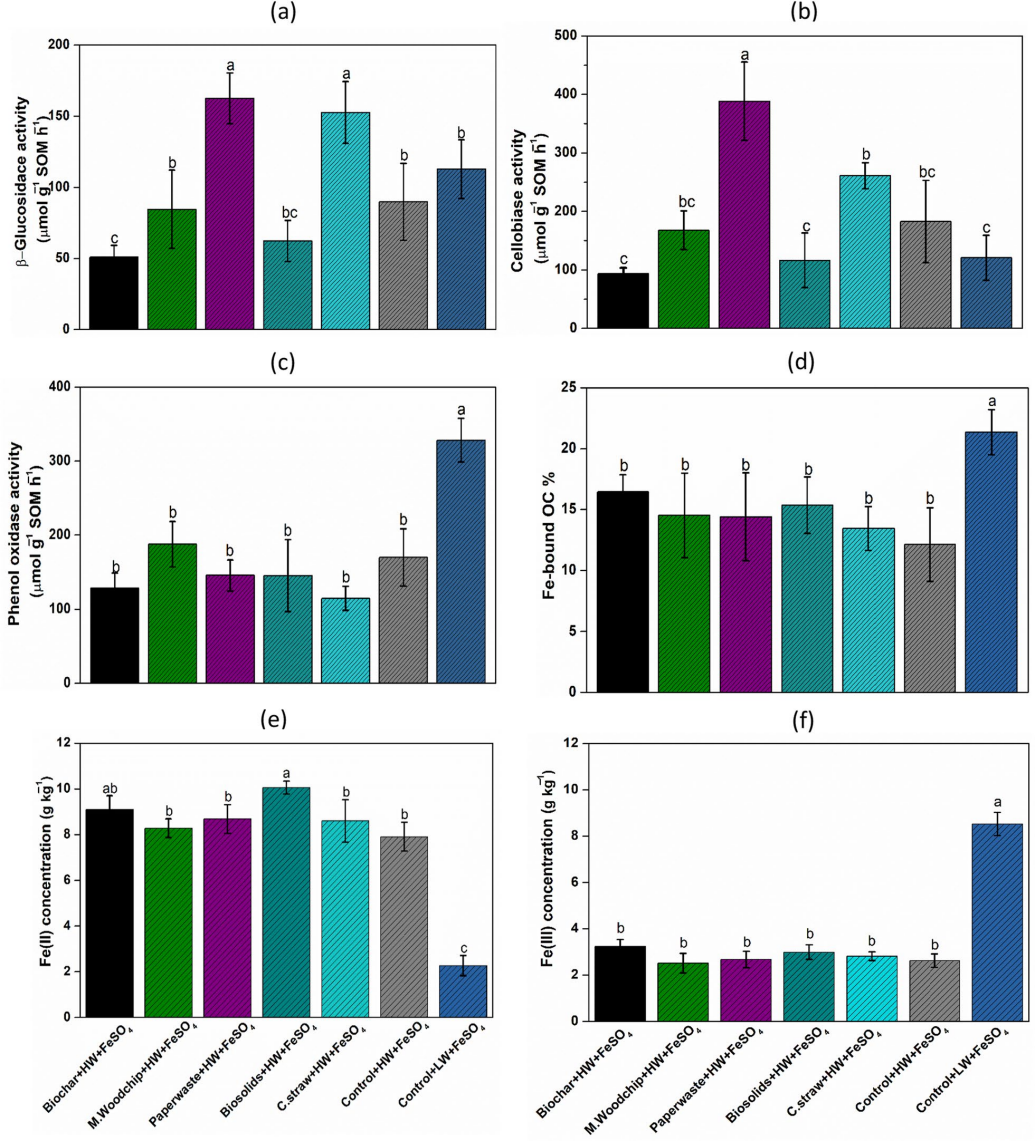
Effect of organic C amendment and FeSO_4_ addition on changes in soil enzyme activities and Fe concentrations in an agricultural peat soil after a year (**a**) β-glucosidase activity; (**b**) cellobiase activity; (**c**) phenol oxidative activity; (**d**) iron-bound soil organic carbon (Fe-bound SOC); (**e**) Fe (II) concentration; (**f**) Fe (III) concentration. The C amendments were loaded at 20 t C ha^−1^ and included biochar (pyrolysed *Miscanthus giganteus* wood chip), commercial paper waste, *M. giganteus* derived chip, barley straw and advanced anaerobically digested biosolids. Water table heights were low water table (LW; − 40 cm, representing no change of management), high water table (HW; 0 cm, representing rewetting) and FeSO_4_ addition ± at a rate of 0.5 t ha^−1^. Values represent mean ± standard error (*n* = 4)

Over the 1-year experiment, soil pH increased in the LW treatment, while biochar addition resulted in a more stable pH of ca. 6.5 during the experimental period (*p* < 0.001; Fig. 1a, b). The Eh of the soil under the low water table regime was relatively stable at + 200 mV throughout the experiment (Fig. 1e, f); however, raising the water table slowly decreased the redox potential for the first 30 d after which it remained relatively uniform for the remainder of the experiment (ca. − 150 mV) in all treatments, irrespective of Fe or organic C addition (Fig. 2e, f; Fig. S1). Soil solution NO_3_^−^ concentrations were significantly higher in the LW treatments relative to those with a high-water table, especially after the addition of FeSO_4_ (Fig. 1g, h). Overall, addition of the organic amendments had no effect on soil NO_3_^−^ concentrations irrespective of FeSO_4_ addition. Soil solution DOC concentration was highest in the biochar + HW and C.straw + HW treatments, while the lowest concentrations were observed in the LW treatment (Fig. S1ab). In the HW treatments, NH_4_^+^ dominated the soluble N pool; however, the concentrations were similar irrespective of amendments and FeSO_4_ addition (Fig. S1cd). All the high-water table treatments had a significantly greater soil solution NH_4_^+^ concentrations relative to the low water table treatment (*p* < 0.001).

The Biochar + HW treatment resulted in the highest aromaticity (8.05 L mg C^−1^ m^−1^) assessed by specific ultraviolet absorbance at 254 nm (SUVA_254_) in soil solution, followed by Biochar + HW + FeSO_4_ (5.2 L mg C^−1^ m^−1^), with the lowest values observed in the Control + LW treatment (1.3 L mg C^−1^ m^−1^) (Fig. S2b).

### Enzyme activity

The highest β-glucosidase and cellobiase activities were observed in the Paperwaste + HW + FeSO₄ treatment, followed by the C.straw + HW + FeSO₄ treatment. In contrast, the Biochar + HW + FeSO₄ and Biosolid + HW + FeSO₄ treatments exhibited the most pronounced declines in the activities of both enzymes (Fig. 2a, b). Specifically, β-glucosidase and cellobiase activities in the Biochar + HW + FeSO₄ were approximately two-fold lower than those in the Control + HW + FeSO₄ treatment.

Phenol oxidase activity, however, showed a different pattern. The Control + LW + FeSO₄ recorded the highest activity at 318 µmol g⁻^1^ SOM h⁻^1^, whereas the Biochar + HW + FeSO₄ treatment exhibited the lowest value at 124 µmol g⁻^1^ SOM h⁻^1^. Phenol oxidase activity was notably influenced by the water table level, with the (LW condition in the Control + FeSO₄ treatment significantly enhancing activity. In contrast, phenol oxidase levels remained relatively unchanged under HW treatments, consistently measuring below 200 µmol g⁻^1^ SOM h⁻^1^.

### Extractable soil Fe concentrations

Soil extractable soil Fe(II) concentration dominated over Fe(III) in the HW treatments, while the extractable soil Fe(III) concentrations significantly increased in the Control + LW + FeSO_4_ treatment (Fig. 2f). Correspondingly, the soluble Fe(III) content was 8 g kg^−1^ in the Control + LW + FeSO_4_, and soil solution Fe(II) concentrations ranged from 8 to 10 g kg^−1^ for all treatments with a high water table at the end of the experiment. Interestingly, Fe bound OC content was greater in Control + LW + FeSO_4_, whereas there was no obvious change found under the high-water table treatment (Fig. 2d).

### Microbial community characteristics

**Fig. 3:**
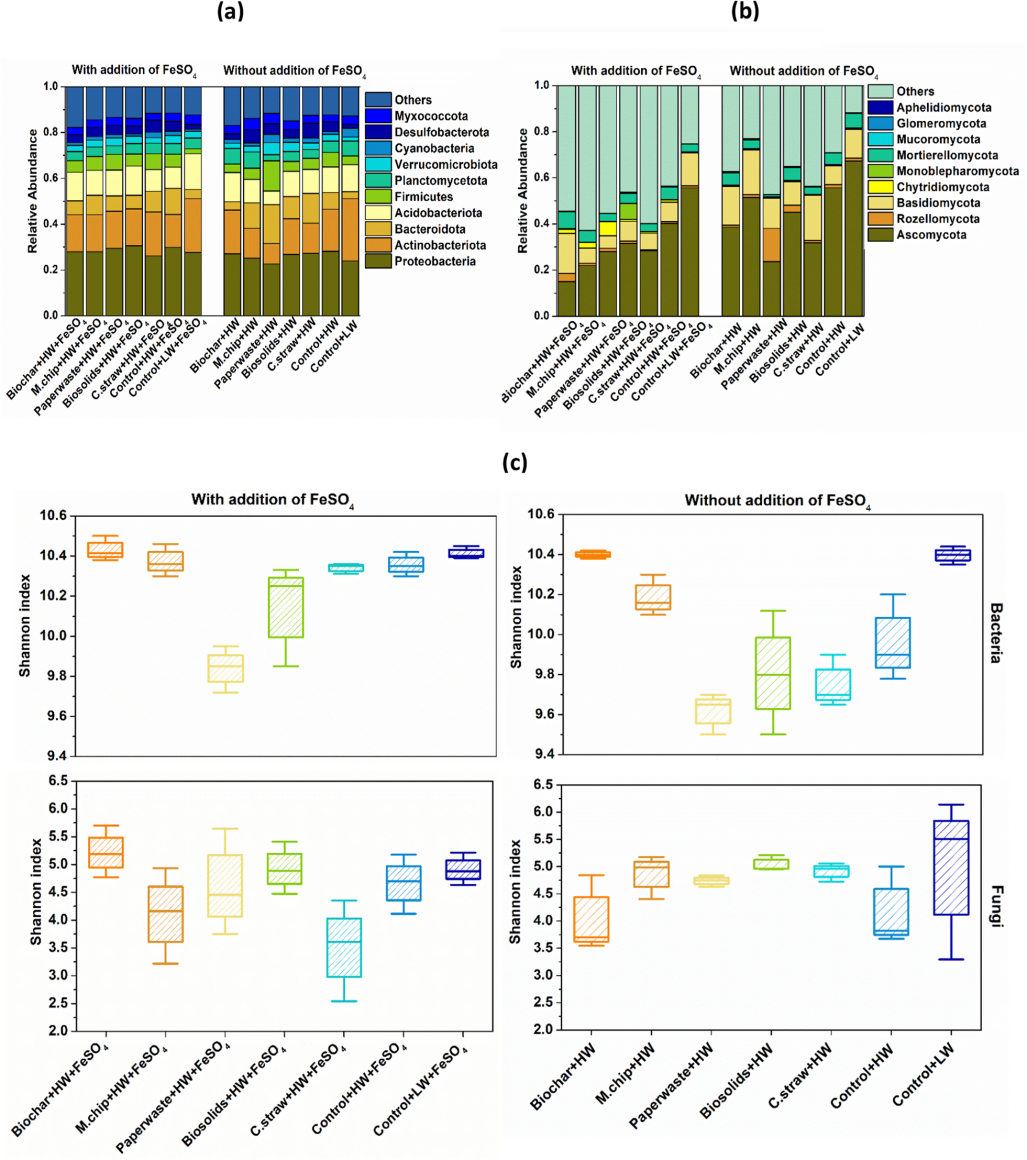

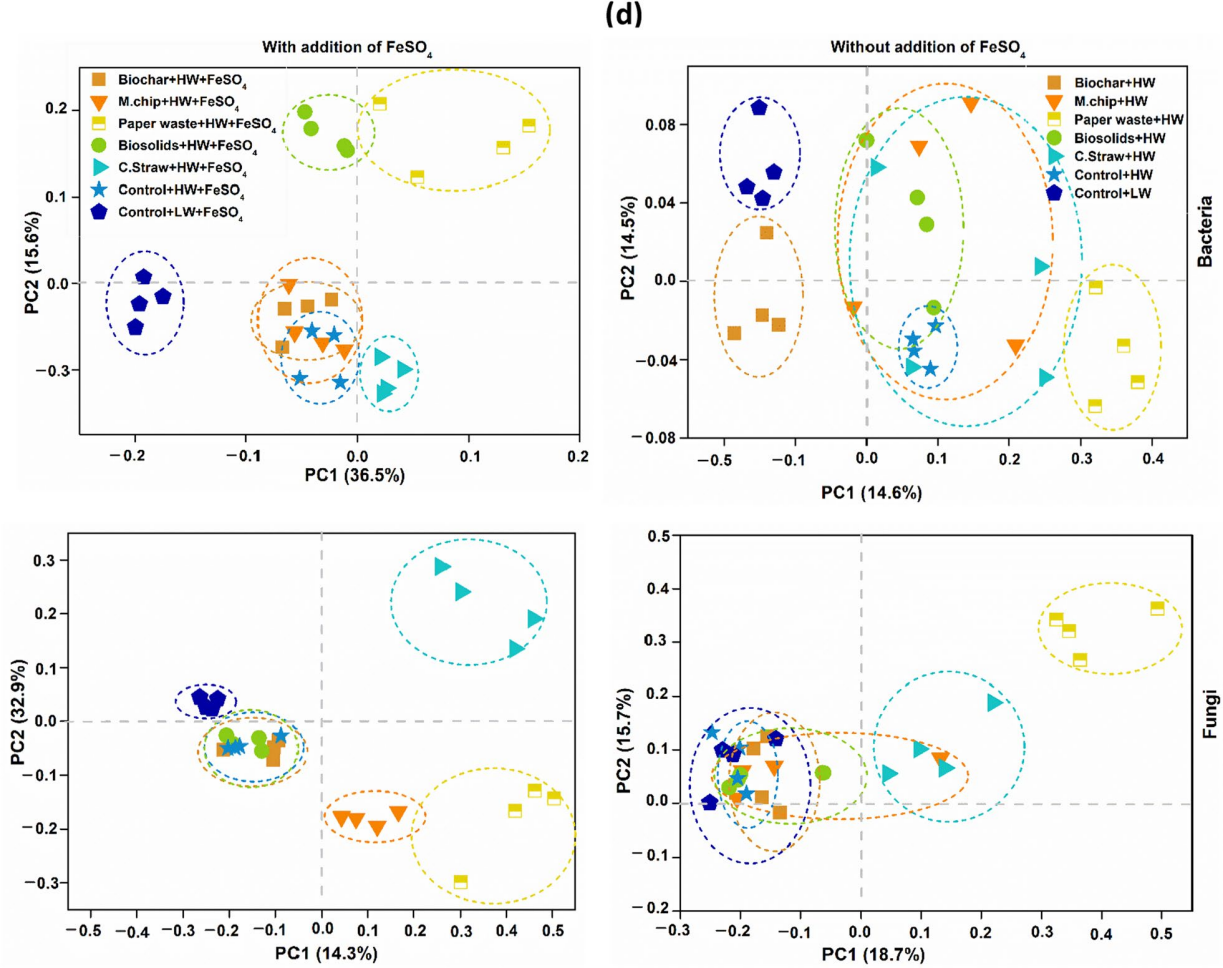
Bacterial and fungal community dynamics in peat mesocosms. **a** and **b** illustrate the relative abundance of bacterial and fungal community, while (**c**) and (**d**) represent alpha diversity and beta diversity of soil bacterial and Fungal communities, respectively. The Shannon index was calculated with all OTUs. The horizontal bars within boxes represent the median. The tops and bottoms of boxes represent 75th and 25th quartiles, respectively. The principal coordinates analysis (PCoA) with Bray–curtis dissimilarity was performed on the taxonomic profile (at the OTU level) of bacterial communities. The C amendments were loaded at 20 t C ha^−1^ and included biochar (pyrolysed *Miscanthus giganteus* wood chip), commercial paper waste, *M. giganteus* derived chip, barley straw and advanced anaerobically digested biosolids. Water table heights were low water table (LW; − 40 cm, representing no change of management), high water table (HW; 0 cm, representing rewetting) and FeSO_4_ addition ± at a rate of 0.5 t ha^−1^. Values represent mean ± standard error (*n* = 4)

The relative abundance of dominant microbial taxa shifted in response to soil amendments and water table depth. Under low water table conditions, *Actinobacteria* prevalence was significantly higher, comprising 16–22% of bacterial communities in controls with or without added FeSO_4_ (Fig. 3a). Correspondingly, *Ascomycota* dominance reached 50–61% in these treatments, declining in other amendment types (Fig. 3b). Microbial diversity based on the Shannon index spanned 9.4–10.5 for bacteria and 2.5–6.4 for fungi across all treatments (Fig. 3c).

Principal Coordinates Analysis (PCoA) was used to further examine the microbial community structure across treatments, revealing distinct shifts associated with the addition of FeSO₄ (Fig. 3d). The bacterial community composition in the Paperwaste + HW + FeSO_4_ and Control + HW + FeSO_4_ treatments was separated from other treatments, whereas the fungal community in the Paperwaste + HW + FeSO_4_ and C.straw + HW + FeSO_4_ showed the largest separation from Control + HW + FeSO_4_ treatments (Fig. 3d). According to the RDA analysis, pH, EC, cellobiase activity, and redox potential had a strong co dependance on both bacterial and fungal community composition (Fig. 5c and d).

Microbial network analysis was performed to discover the interactive relationships between bacterial and fungal communities across treatments. The ratio of positive associations (co-presence) to negative associations (mutual exclusion) was highest in the Control + LW + FeSO₄ treatment and lowest in the Control + HW + FeSO₄ treatment for both microbial groups (Table 1). The fungal network of the Control + LW + FeSO_4_ treatment represented 73.1% of nodes affiliated with *Ascomycota,* with the genera *Scutellinia* and *Pyrenochaetopsis* detected as keystone taxa (Table 1). Changes in bacterial and fungal community composition across four treatments were further assessed using Linear Discriminant Analysis Effect Size (LEfSe) (Segata et al. 2011). This approach, applied from the phylum to genus level, enabled the identification of differentially abundant taxa while accounting for biological consistency and effect size. LEfSe results highlighted specific microbial lineages strongly associated with individual treatments (Fig. 3b), including a notable enrichment of methanogenic genera such as *Methanosarcina* in the Paperwaste + HW treatment.

**Table 1:** Properties of bacterial and fungal co-occurrence networks

Properties	Biochar + HW + FeSO_4_			Control + HW + FeSO_4_		Control + LW + FeSO_4_		
	Bacteria	Fungi		Bacteria	Fungi	Bacteria		Fungi
Nodes	170		76	151	69	201		82
Edges	135		69	122	150	164		72
Modularity	0.879		0.835	0.742	0.604	0.807		0.5
ACC	0.121		0.099	0.132	0.200	0.145		0.164
APL	1.181		1.394	1.142	1.644	1.164		1.145
Copresence	82		72	87	84	83		55
Mutual exclusion	18		27	13	16	17		45
Ratio of positive to negative links	4.8		2.6	6.6	5.6	4.5		1.2
Proteobacteria (%)	30.3		34.2	33.7	56.6	30.3		73.1
Actinobacteria (%)	25.8		5.2	18.5	5.8	22		6.1
Acidobacteria (%)	16.9		52.6	17.8	31.8	13		15
Keystone taxa	*Pedomicrobium* *Solirubrobactor*		*Scutellinia*	*Anaeromyxobactor* *ADurb.Bir063-1*	*Acremonium*	*Nocardioides*	*Pyrenochaetopsis*	

### Relationships between measured environmental variables and microbial community composition

Distance-based linear model (DistLM) analysis indicated that bacterial community composition was influenced by redox potential (9.2% explanatory power), phenol oxidase activity (6.2%), β-glucosidase activity (7.7%), and total Fe (5.2%). The fungal community was affected by NO_3_^−^ concentration (10.6% explanatory power), phenol oxidase activity (8.1%), total Fe (6.5%), NH_4_^+^ concentration (5.0%), α-glucosidase activity (4.5%) and β-glucosidase activity (4.7%) (Table 2). Total soil Fe and phenol oxidase activity contributed to shifts in both bacterial and Fungal communities, explaining 11.7% and 14.3% of community variation, respectively (Table 2).

**Table 2:** Contributions of soil edaphic variables to shaping the bacterial and fungal community based on Bray–Curtis dissimilarities analyzed by distance-based linear modeling (distLM) analysis

Soil edaphic variables	Contribution to bacteria %	Soil edaphic variables	Contribution to fungi %
Redox potential	9.2*	NO_3_^−^	10.6***
Phenol oxidase activity	6.2*	Phenol oxidase activity	8.1***
α-glucosidase activity	7.7**	Total Fe	6.5***
Total Fe	5.2*	NH_4_^+^	5.0**
β-glucosidase activity	4.5	α-glucosidase activity	4.5*
pH	3.4	β-glucosidase activity	4.7*
NH_4_^+^	3.5	Temperature	3.4
Total dissolved N	3.7	pH	3.2
DOC	4.8	Total dissolved N	3.2
Temperature	2.6	DOC	3.3

*, ** and *** indicate a significant result at the *p* < 0.05, *p* < 0.01 and *p* < 0.001 level, respectively

Correlation analysis further showed that bacterial community structure was significantly related to phenol oxidase activity, pH, cellobiase activity and DOC (Mantel’s *r* ≥ 0.2, *p* < 0.01), while the fungal community correlated with DOC, NO_3_^−^, Fe, and enzyme activities (Mantel’s *r* < 0.2 to 0.4, *p* < 0.01 to 0.05) (Fig. 5). Co-occurrence networks demonstrated that ratios of positive to negative links between microbes were highest in the Control + LW + FeSO_4_ treatment, and fungal nodes were dominated by Ascomycota (73.1% of nodes) for which *Scutellinia* and *Pyrenochaetopsis* were identified as keystone genera (Table 1).

## Discussion

### Effects of water table management on soil solution chemistry

During the 365-day experimental period, temporal variability of concentration of NO_3_^−^ was significantly higher in the Control + LW + FeSO_4_ treatment compared to all other treatments. The accumulation of NO₃⁻ observed in this study is likely attributed to aerobic nitrification processes, whereby soil organic matter (SOM) is mineralized, leading to the conversion of organic nitrogen into inorganic forms and the subsequent release of nitrate. Our findings indicate that nitrification predominated under well-aerated conditions, whereas denitrification became the primary nitrogen transformation pathway in treatments characterized by high moisture and anaerobic conditions (Zhu et al. 2013; Huang et al. 2014). These patterns align with previous research on pH-neutral, lowland peat soils, which has shown that lowering the water table promotes nitrification and associated N₂O emissions (Taghizadeh-Toosi et al. 2019), while raising the water table suppresses these processes (Taft et al. 2018). This contrasts with acid upland peat soils where the low soil pH limits can inhibit nitrification (Marsden et al. 2019) were highest among all the treatments, indicating that applying biochar to soil generally leads to an increase in the soil’s DOC content, which means that biochar can raise the level of organic C readily available in the soil solution (Nakhavali et al. 2020). This is primarily due to the high C content of biochar itself, and its ability to adsorb and retain organic matter within its porous structure (particularly prevalent in lignocellulosic structures such as *Miscanthus*). After 90 days, the DOC content declined (from 500 to 190 mg L^−1^ soil in FeSO_4_-amended treatment), indicating that leaching of DOC through the mesocosm or sorption of native soil DOM by biochar likely occurred during the experiment period, which might be due to leaching of DOC through the soil profile and the high sorption capacity of biochar for soil DOC (Kasozi et al. 2010; Nakhavali et al. 2020). Additionally, an increase in soil dissolved organic carbon (DOC) content was noted following the application of 500 °C biochar derived from rice straw (Yang et al. 2022a) and wheat straw (Zhang et al. 2017b; Zhang et al. 2021b). These varying outcomes may be attributed to differences in the intrinsic properties of the biochar, which are influenced by feedstock type and pyrolysis conditions. SUVA₅₂₄ values used as a proxy for the aromaticity of DOM are shown in Fig. S2bc, ranging from 5.2 to 8 L mg C⁻^1^ m⁻^1^. Both biochar treatments, with and without FeSO₄ addition, significantly (*p* < 0.05) elevated SUVA₅₂₄ values compared to the control, indicating enhanced aromaticity of soil DOM (Yang et al. 2021).

During the 365-day experimental period, NO₃⁻ concentrations exhibited significantly greater temporal variability in the Control + LW + FeSO₄ treatment compared to all other treatments. This pronounced NO₃⁻ accumulation is best explained by aerobic nitrification of SOM, where microbial oxidation of organic nitrogen results in the release of NO₃⁻. Under low moisture and oxic conditions, nitrification appeared to be the dominant nitrogen transformation pathway. In contrast, treatments with higher water tables created more anaerobic conditions, which favored denitrification as the primary nitrogen pathway (Zhu et al. 2013; Huang et al. 2014; Jeewani et al. 2025). These findings are supported by prior studies in lowland, pH-neutral peat soils, where lowering the water table increased N₂O emissions via enhanced nitrification, while raising the water table suppressed these emissions (Taghizadeh-Toosi et al. 2019; Taft et al. 2018). In contrast, nitrification is less prominent in acid upland peat soils due to pH limitations (Marsden et al. 2019).

These results support the hypothesis that nutrient stoichiometry specifically the balance between carbon and nitrogen availability modulates microbial activity and decomposition processes in peat soils. Under saturated (anaerobic) conditions, microbial access to oxygen is restricted, and nitrogen becomes a co-limiting factor, particularly when high C:N ratio substrates (e.g., Biochar + HW) are present. In contrast, under aerobic conditions and with lower C:N ratios, nitrogen availability increases, stimulating nitrifier populations and associated enzyme activities. Thus, the data reveal a mechanistic link between hydrological conditions, nutrient availability, and microbial nitrogen cycling, validating the hypothesis that stoichiometric constraints regulate microbial functioning in peatland environments.

Moreover, DOC concentrations were highest in the biochar-amended treatments (with and without FeSO₄), highlighting biochar’s capacity to elevate dissolved organic carbon in soil solutions. This increase can be attributed to biochar's high C content and its porous structure, which allows for adsorption and retention of both added and native organic matter (Nakhavali et al. 2020). However, DOC levels declined after 90 days (e.g., from 500 to 190 mg L⁻^1^ in Fe-amended treatments), likely due to leaching losses and sorption of native dissolved organic matter (DOM) by biochar (Kasozi et al. 2010). The observed differences across studies (e.g., Yang et al. 2022b; Zhang, et al., 2017a); Zhang, et al. 2021a) may reflect variations in feedstock type and pyrolysis conditions, which influence the physicochemical properties of biochar such as total organic carbon, aromaticity, and porosity pore connectivity (Keiluweit et al. 2010; Kloss et al. 2012).

Additionally, both biochar treatments significantly increased SUVA₂₅₄ values (ranging from 5.2 to 8 L mg C⁻^1^ m⁻^1^), an indicator of DOM aromaticity (Fig. S2bc), suggesting that biochar enhanced the aromatic character of soil DOC (Yang et al. 2022b). These changes further support the view that biochar influences not only carbon quantity but also carbon quality in peat systems, with potential implications for microbial processing and carbon stability.

### Microbial responses to a change in water levels and organic amendments

Bacteria and fungi play distinct but complementary roles in peat carbon decomposition (Kluber et al. 2020). In our study, variations in microbial abundance and composition were strongly driven by changes in water table levels and amendment quality, emphasizing the influence of C resource traits on microbial ecological strategies and peat decomposition. Microbial abundance patterns confirmed that LW conditions favored aerobic metabolism, as reflected in significantly increased bacterial and fungal populations (Fig. 3a, b). The highest abundance of *Actinobacteria*, observed in the Control + LW treatments regardless of FeSO₄ addition (Fig. 2), suggests that dry, oxic environments with relatively labile organic matter support r-strategist bacterial taxa capable of rapid resource exploitation(Richy et al. 2024). These bacteria are also known to stimulate SOC mineralization via microbial priming, due to their mycelial morphology and ability to access otherwise protected C pools (Luo et al. 2017; Fu et al. 2022). This coincided with the highest recorded CO₂ emissions (17.4 t CO₂e ha⁻^1^ yr⁻^1^; Jeewani et al. 2025), likely the result of enhanced microbial turnover and accelerated carbon cycling (Luo et al. 2011).

**Fig. 4:**
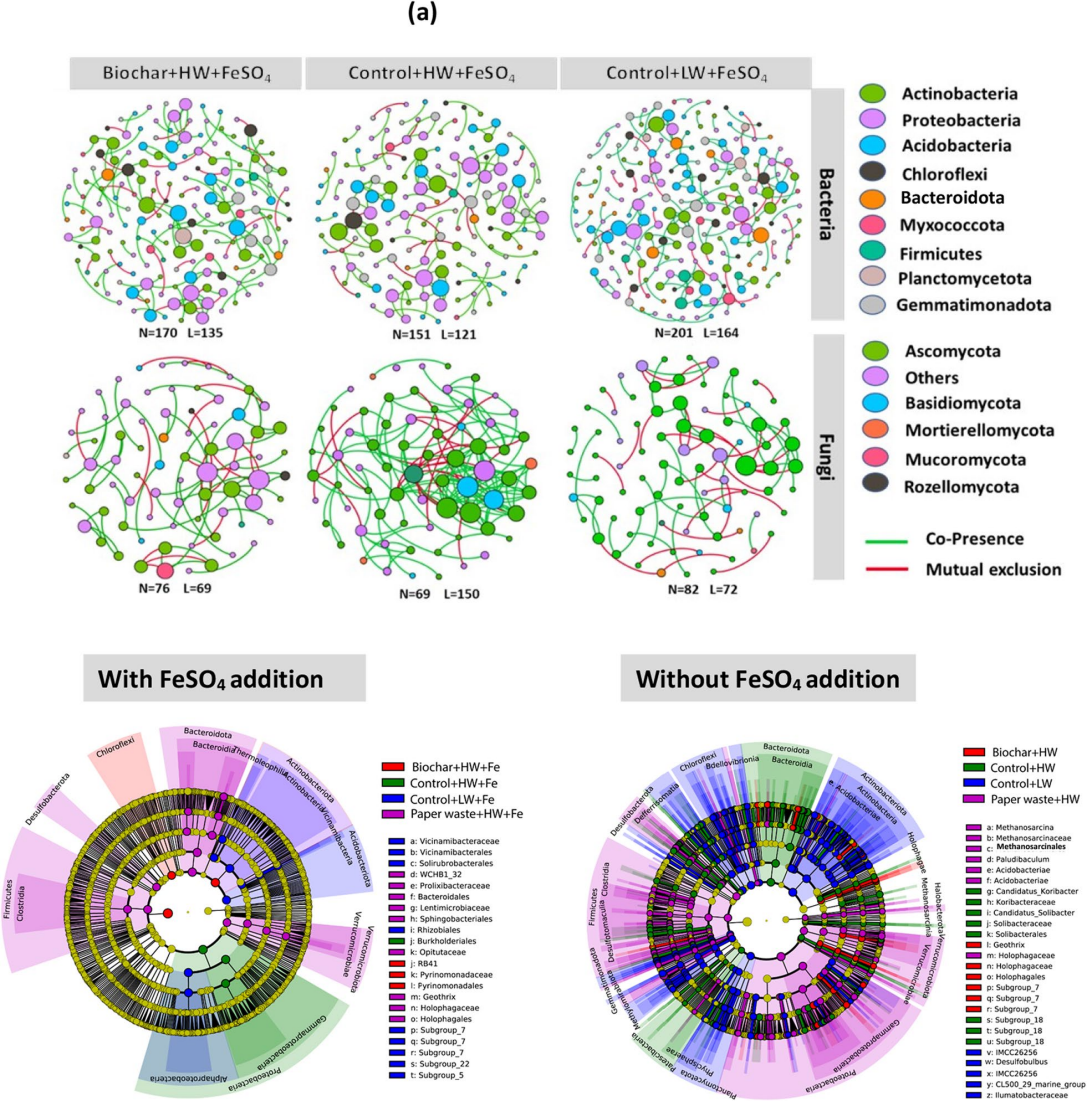
Co-occurrence networks of the abundance OTU (**a**) N, node. L, line. Edges represent significant Spearman correlations (ρ >|0.8|, *p* < 0.05). Red Lines represent a significant negative correlation, and green Lines represent a significant positive. Changes in bacterial community composition between the selected 4 treatments out of all treatments (Linear discriminant analysis Effect Size) explain differences between classes by coupling standard tests for statistical significance with additional tests encoding biological consistency and effect relevance (**b**). The C amendments were loaded at 20 t C ha^−1^ and included biochar (pyrolysed *Miscanthus giganteus* wood chip), commercial paper waste, *M. giganteus* derived chip, barley straw and advanced anaerobically digested biosolids. Water table heights were low water table (LW; − 40 cm, representing no change of management), high water table (HW; 0 cm, representing rewetting) and FeSO_4_ addition ± at a rate of 0.5 t ha^−1^. Values represent mean ± standard error (*n* = 4)

Fungal community shifts further supported the resource-quality-driven life history response. Ascomycota, a phylum composed largely of K-strategists with high substrate versatility and stress tolerance, dominated the Control + LW treatment (68% relative abundance). These fungi were significantly associated with oxidative enzyme activity especially phenol oxidase (8.1; *p* < 0.01; Fig. 4, Table 2) indicating their role in degrading aromatic, complex organic matter. The microbial network in this treatment was also dominated by Ascomycota (73.1%), including cellulolytic keystone genera such as *Scutellinia* and *Pyrenochaetopsis*, known to contribute to the mineralization of stable C compounds (Zhang et al. 2021a). This dominance aligns with increased CO₂ flux and further confirms that K-strategist fungi are selectively enriched in conditions where C availability is high but structurally complex (Yi et al. 2019; Boer et al. 2005).

In contrast, saturated, HW conditions especially in the Paper waste+HW treatment favored anaerobic C pathways. CH₄ emissions were significantly elevated (Table 1; Jeewani et al. 2025), coinciding with increased abundance of the acetoclastic methanogen *Methanosarcina* (Fig. 4b). This suggests that under reducing conditions, r-strategist methanogens exploit labile C inputs from cellulose-rich amendments. Supporting studies have shown that acetate-using (*Methanotrichaceae*) and hydrogenotrophic (*Methanobacteriaceae*) methanogens dominate methane production in organic-rich peat systems (Breeuwer et al. 2009; Corbett et al. 2015; Bräuer et al. 2020).

**Fig. 5:**
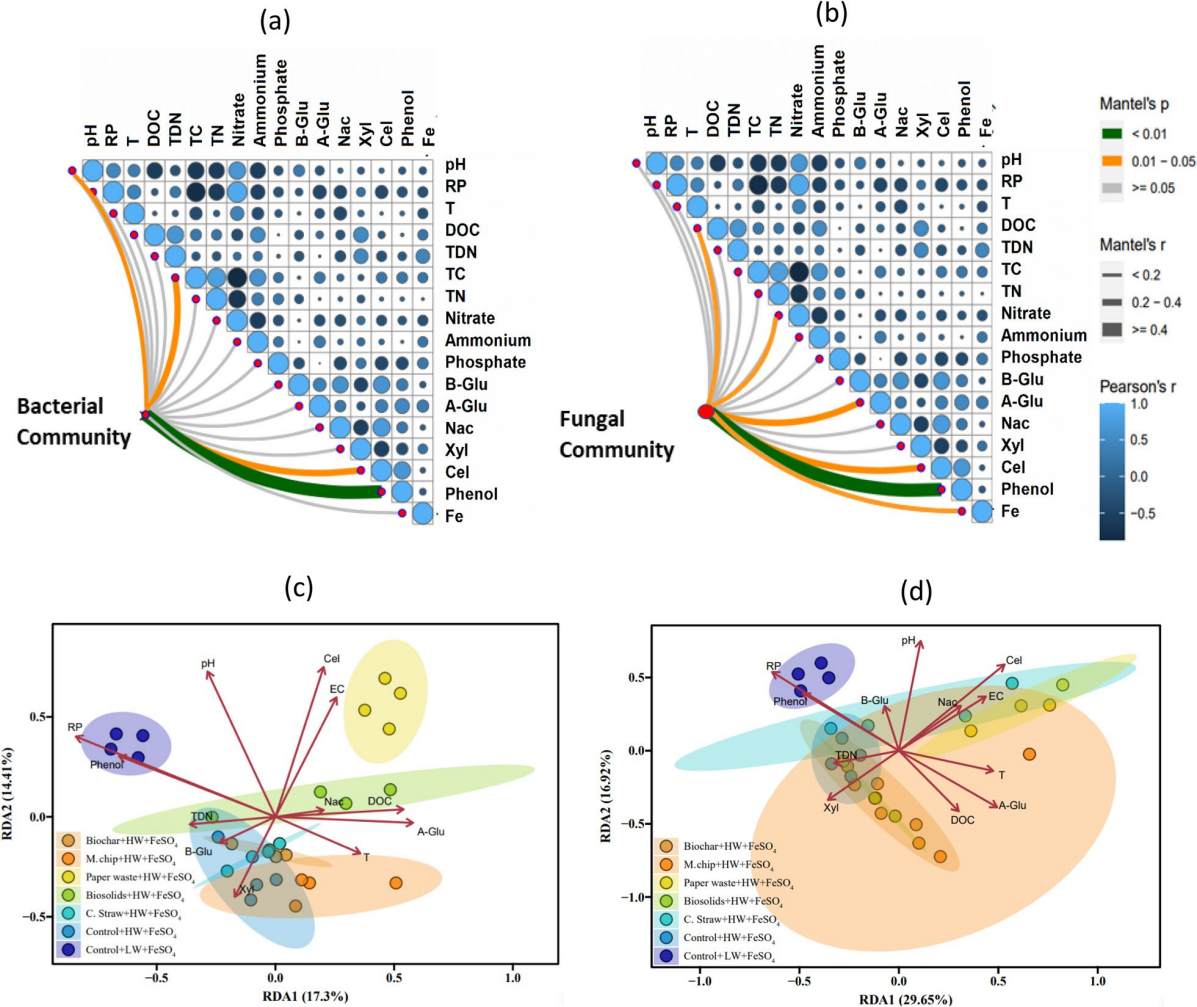
Correlations of the bacterial and fungal community composition (Bray–Curtis distance) with soil variables (**a** and **b**, respectively). Edge width corresponds to the Mantel’s *r* value, and the edge colour denotes the statistical significance. Pairwise correlations of these variables are shown with a colour gradient denoting Pearson’s correlation coefficient. **c** and **d** represent redundancy analysis (RDA) showing the relationship between the soil bacterial (**e**) and fungal (**f**) community and environmental factors. Abbreviation for soil variables include pH, RP, redox potential; T, Temperature; DOC, dissolved organic carbon; TDN, total dissolved nitrogen; TC, total carbon; TN, total nitrogen; Nitrate; NO_3_^–^, Ammonium; NH_4_^+^, phosphate; PO_4_^3−^, B-Glu; β-glucosidase, Nac; N-acetyl-β lucosaminidases, Xyl; β-Xylosidases, Cel; β-Cellobiosidase, Phenol; phenol oxidase and Fe; total Fe concentration. The C amendments were loaded at 20 t C ha^−1^ and included biochar (pyrolysed *Miscanthus giganteus* wood chip), commercial paper waste, *M. giganteus* derived chip, barley straw and advanced anaerobically digested biosolids. Water table heights were low water table (LW; − 40 cm, representing no change of management), high water table (HW; 0 cm, representing rewetting) and FeSO_4_ addition ± at a rate of 0.5 t ha^−1^. Values represent mean ± standard error (*n* = 4)

Biochar application had a marked effect on microbial life strategies and ecosystem-level C and N cycling. In biochar-amended soils, the relative abundance of Ascomycota declined significantly, and microbial co-occurrence networks were simplified (Figs. 3a–d, 4a), indicating that biochar altered the ecological niches available to K-strategist fungi. This shift likely reflects changes in DOC quality particularly reductions in labile C and increases in aromatic C resulting from biochar’s sorptive capacity and structural properties (Zimmerman 2010; Han et al. 2016). The “charosphere” environment also promoted microbial carbon use efficiency and moderated community composition (Quilliam et al. 2013; Lehmann et al. 2011), favoring generalist taxa and possibly enhancing nutrient immobilization (Fig. 5).

Functionally, biochar significantly suppressed CH₄ emissions by 20.7 t CO₂e ha⁻^1^ yr⁻^1^ (Jeewani et al. 2025), likely through inhibition of methanogenic activity. This suppression was linked to a lower ratio of methanogen (mcrA gene) to methanotroph (pmoA gene) abundance (Dong et al. 2013; Han et al. 2016), a pattern consistent with reduced availability of easily fermentable substrates. Thus, biochar appears to act as a selective filter, modifying resource quality and shaping microbial community assembly and function in ways that suppress r-strategist anaerobes and alter K-strategist fungal dominance.

Furthermore, nitrifying microbial genera (e.g., *Nitrosospira*, *Nitrosomonas*, *Nitrosovibrio tenuis*, *Bacillus* spp.) were dominant under low water table conditions (Fig. 4b, Table 2), corresponding with high NO₃⁻ availability and N₂O emissions (Jeewani et al. 2025). These taxa, adapted to oxic, nutrient-rich environments, exemplify r-strategist behavior by rapidly exploiting available NH₄⁺ and promoting nitrification-driven N losses. The DistLM model corroborated these findings, showing strong associations between microbial community shifts and environmental variables such as redox potential (9.2; *p* < 0.01) and NO₃⁻ content (10.6; *p* < 0.001).

Collectively, these results confirm that resource quality not just its quantity plays a pivotal role in structuring microbial life strategies in peat soils. Under nutrient-rich and oxic conditions, r-strategists dominate and promote rapid mineralization, while biochar moderates these responses by constraining labile resource availability and selectively shaping community composition. These insights offer a mechanistic basis for understanding how peatland management strategies such as biochar addition can modulate decomposition pathways and greenhouse gas emissions by altering microbial resource use strategies.

### Trade-offs between the ‘enzyme latch’ and ‘iron gate’ mechanisms

Soil enzymes play a main role in SOM decomposition (Soares and Rousk 2019). The soil enzyme activities among organic amendments, as well as water table levels, differed significantly (Fig. 2a–c), indicating that they were significantly affected by availability of organic C and hydrological management practices (Fig. S4). Hydrolytic enzyme activities (β-glucosidase and cellobiohydrolase) were significantly higher in the Paper Waste + HW + FeSO₄ and C.straw treatments compared to the Control + HW + FeSO₄ treatment. This enhancement may be attributed to the substantial increase in available carbon substrates for microbial mineralization. Previous studies also reported lower soil enzyme activities in degraded agricultural peatland with reduced C source than those in natural peatland, thus limiting soil enzyme activity (Wang et al. 2021).

However, phenol oxidative activity increased in the low water table peat mesocosms upon exposure to O_2_ (Freeman et al. 1996, 2001b). In contrast, several studies have observed that the presence of Fe(II) in hypoxic peatland soils may enhance phenol oxidative activity (Li et al. 2012; Hall and Silver 2013). However, the activity of polyphenol oxidase and Fe(III) content in Control + LW + FeSO_4_ was higher than in other treatments (Fig. 2c). This may be due to the low soil water content and good soil air permeability in the soil core, which are favorable to the oxidation of Fe(II) and the survival and reproduction of aerobic microorganisms, thereby promoting degradation of phenolic compounds and facilitating SOM decomposition (Freeman et al. 1996, 2004; Romanowicz et al. 2015). This finding indicates that oxygen availability is the primary factor influencing phenol oxidase activity in this study.

In contrast, HW treatments resulted in reducing conditions with low phenol oxidase activity. These results are consistent with the mechanism associated with the “enzyme latch” from previous studies (Freeman et al. 2001a; Fenner and Freeman 2011; Wang et al. 2024b).

Fe(II) oxidation plays a key role in biochemical processes during water table fluctuation in peat soils that involves the mobilization and stabilization of C (Li et al. 2012; Wang et al. 2024b). It is noteworthy that Fe(III) concentration and Fe bound OC were greater in the Control + LW + FeSO_4_ treatment cores (*p* > 0.05; Fig. 2d), which is consistent with previous publications regarding the “iron gate” mechanism (Wang et al. 2017). In drained peat cores, soluble Fe(II) is oxidized to less soluble Fe(III), enhancing SOM preservation. Furthermore, SOM may absorb or co-precipitate with the newly-formed reactive Fe(III) oxides, forming Fe-bound OC complexes that are chemically more stable (Kaiser and Guggenberger 2007; Riedel et al. 2013; Feng et al. 2025). Such associations between iron and carbon are thought to play a key role in stabilizing and preserving soil C (Kaiser and Guggenberger 2007; Lalonde et al. 2012). In particular, aromatic and phenolic compounds that accumulate in wetland environments have been shown to bind strongly to iron oxides (Lalonde et al. 2012; Feng et al. 2025), potentially protecting them from decomposition in the presence of O_2_ (Hall et al. 2016). Full quantification of C balance in soil cores with low and high-water table levels offers the unique opportunity to explore the trade-offs between the “enzyme latch” and “iron gate” mechanisms.

### Implications

**Fig. 6:**
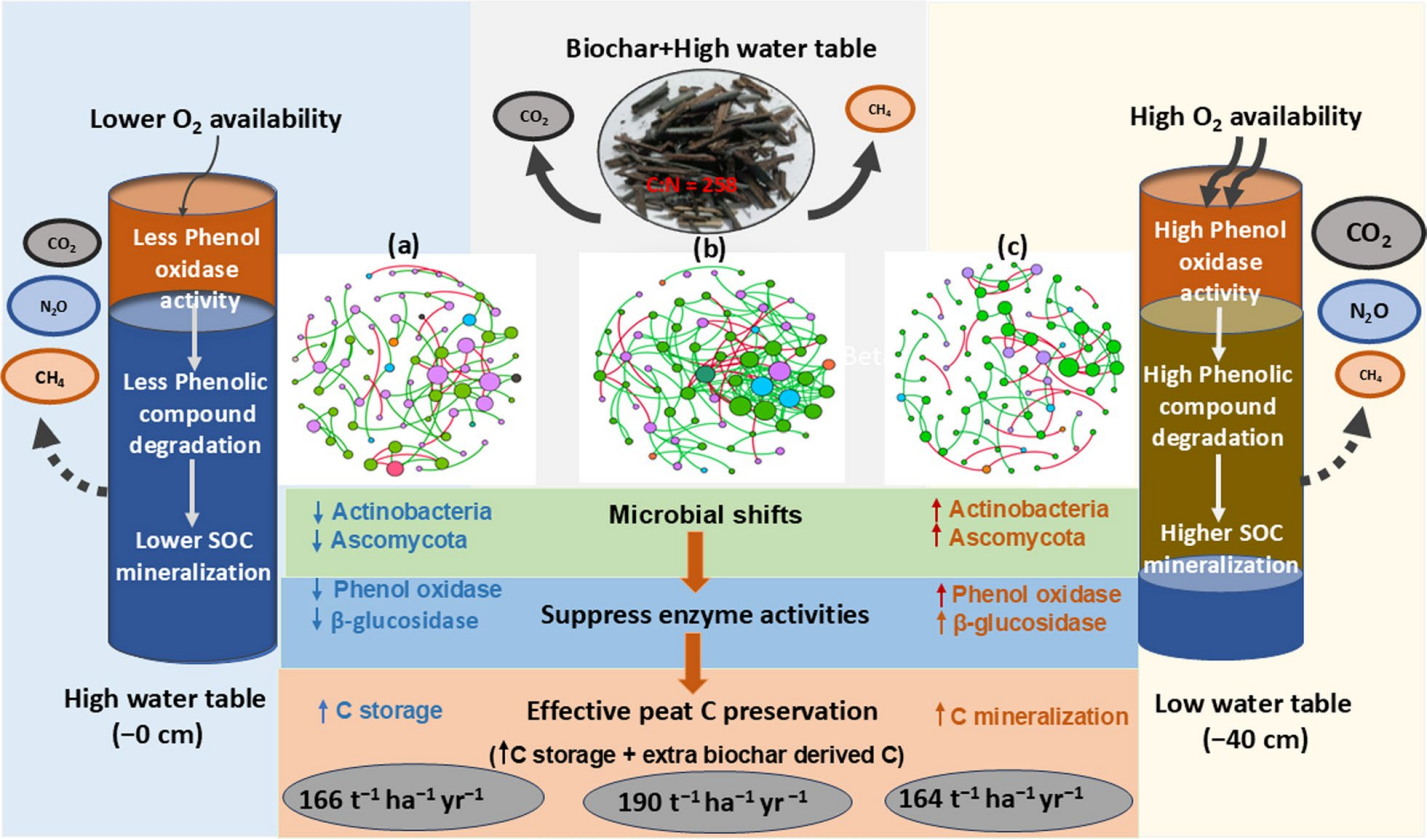
Proposed mechanisms for microbially mediated of soil organic carbon (SOC) preservation in peat soil alongside with rewetting and biochar amendments after 360 days of mesocosm experiment. Significant difference in C pools between three main treatments such as Control + high water table, Control + low water table and Biochar + high water table *p* < 0.05. **a, b and c** indicate fungal co-occurrence networks of each treatment and its complexity (nodes represent different phylum and lines represent negative (green) and positive (red) interactions). Numbers indicate new C retention in each pool based on Jeewani et al. (2025)

Sustainable management strategies that increase C stocks while simultaneously reducing GHG emissions are needed to restore drained peatlands. Large-scale rewetting is currently regarded as the most effective strategy for halting further degradation and subsidence of peat soils, while also mitigating GHG emissions—particularly CO₂ (Evans et al. 2021). While rewetting is a critical step in the ecological recovery of drained peatlands, its success in reducing overall GHG emissions is strongly influenced by the interplay between soil geochemistry and resident microbial communities. Sustaining a high-water table in agricultural peatlands tends to enhance the abundance and potentially the activity of methanogenic microbes, which can in turn result in increased CH₄ emissions (Jeewani et al. 2025). The presence of terminal electron acceptors such as Fe(III) and SO_4_^2−^ inhibits methanogenesis. Oxic conditions promote nitrification by enhancing the activity of nitrifying bacteria, which can result in increased short-term N₂O emissions depending on the geochemical characteristics and microbial community structure (Wang et al. 2024a). The modified microbial abundance, diversity and co-occurrence pattern towards reduced abundance of C decomposers in the Biochar + HW + FeSO_4_ treatment suggests that this combination of treatments shows significant potential for increasing the C stabilised in the peat, through the suppression of CH_4_ emissions in addition to adding recalcitrant C (Fig. 6). As such it is highly recommended that such amendments should be trialed at a larger field scale over longer timeframes.

## Conclusion

This study investigated the biogeochemical dynamics of agricultural peat soils under various organic and inorganic amendment strategies alongside with water table management. Our results demonstrate that maintaining a high-water table combined with biochar is a critical factor in suppressing key decomposer microbial taxa, particularly Ascomycota and Actinobacteria, along with their associated enzymatic activities. Amendments with low C:N ratios such as biosolids, straw and paper waste significantly promoted r-strategist microbial populations by alleviating limitations in labile carbon and nitrogen availability and altering microbial community composition. Notably, the combination of an elevated water table and paper waste enhanced methanogenic activity, while the application of high C:N biochar under the same conditions suppressed Ascomycota abundance. Moreover, the integrated application of biochar, an elevated water table, and FeSO₄ (Biochar + HW + FeSO₄) substantially restructured fungal and bacterial co-occurrence networks via modified biogeochemistry of peat soil. Collectively, these findings suggest that the Biochar + HW + FeSO₄ treatment regime effectively alters microbial interactions and biogeochemical processes, contributing to reduced organic matter decomposition and GHG emissions. This integrated management approach offers a promising strategy for enhancing the sustainability and climate resilience of agricultural peatlands.

## Supplementary Information

Additional file 1.

## Data Availability

Data available within the article or its supplementary materials.
