# Supplementary material for: Restoring degraded agricultural peatlands: how rewetting, biochar, and iron sulphate synergistically modify microbial hotspots and carbon storage
Source: Biochar. 2025 Sep 10;7(1):108. doi: 10.1007/s42773-025-00501-y (PMC12423214; doi:10.1007/s42773-025-00501-y)
Supplement: Supplementary file 1 — Additional file 1. [file 42773_2025_501_MOESM1_ESM.docx]

**Supplementary information**

**Restoring degraded agricultural peatlands: How rewetting, biochar, and iron sulphate synergistically modify microbial hotspots and carbon storage**

Peduruhewa H. Jeewani^a, *^, Robert W. Brown^a^, Jennifer M. Rhymes^b^, Chris D. Evans^b^, Dave R. Chadwick^a^, Davey L. Jones^a^

^a^ *School of Environmental and Natural Sciences, Bangor University, Bangor, Gwynedd, LL57 2UW, UK*

^b^ *UK Centre for Ecology and Hydrology, Bangor, Gwynedd, LL57 2UW, UK*

^*^Corresponding author: Peduruhewa H. Jeewani

Corresponding Author Address: School of Environmental and Natural Sciences, Bangor University, Gwynedd, LL57 2UW

Corresponding Author Email: [j.hemamali@bangor.ac.uk](mailto:j.hemamali@bangor.ac.uk)

**Fig. S1**

(a)

(b)

(c)

(d)


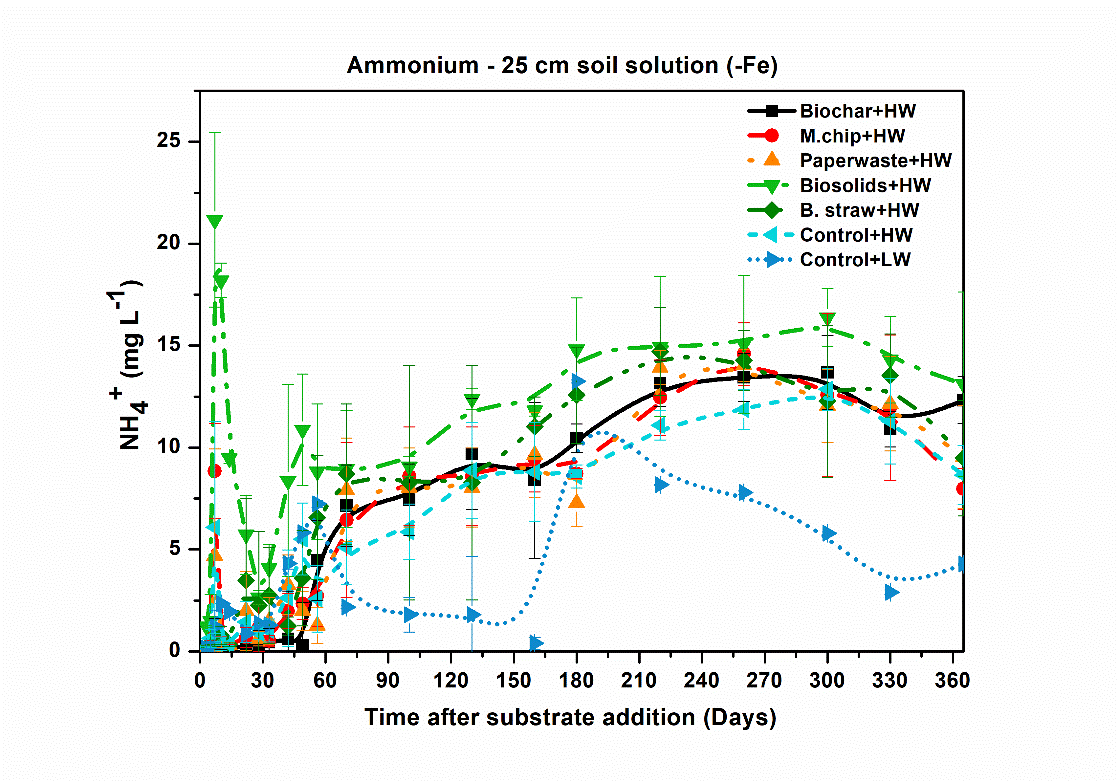

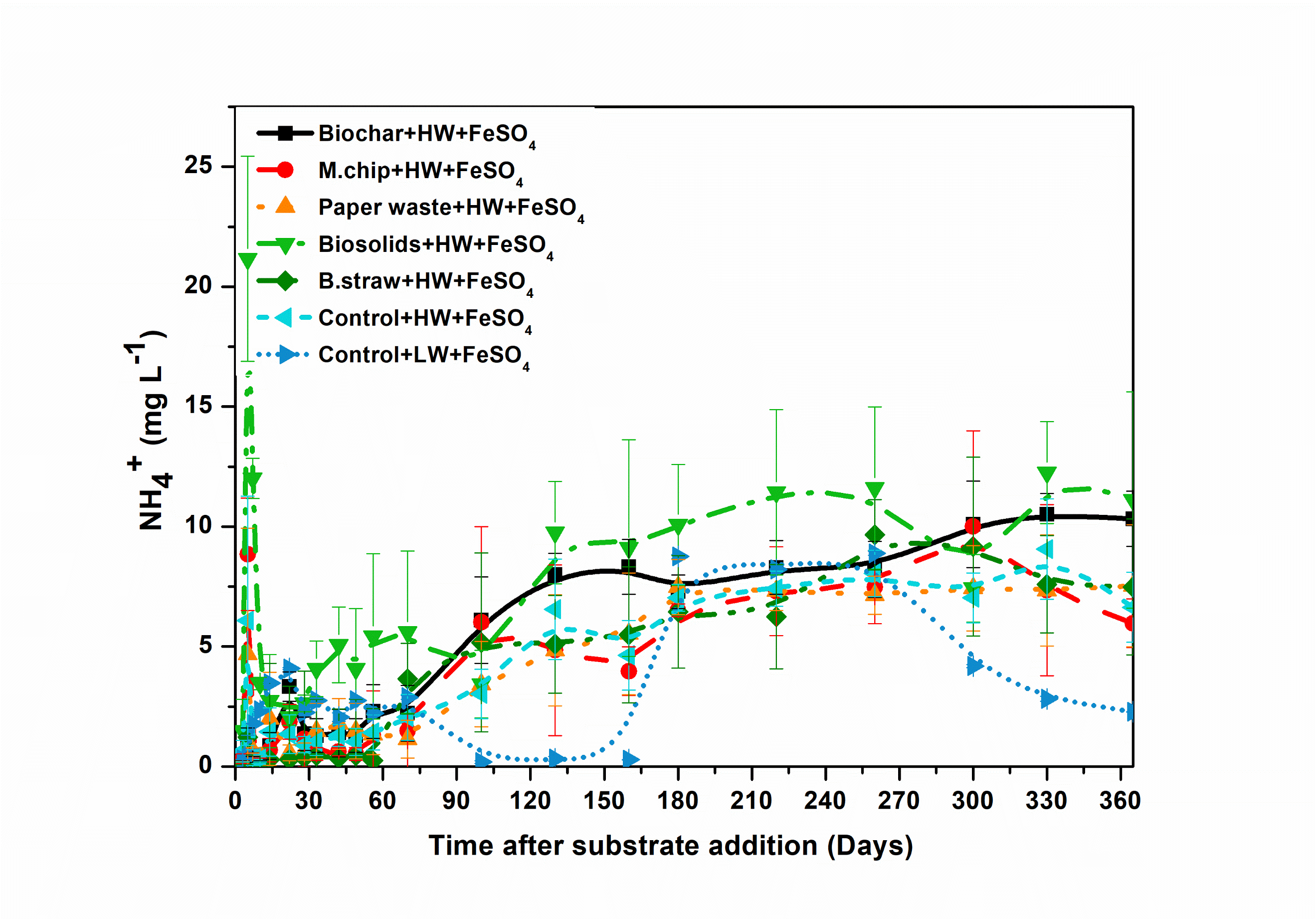

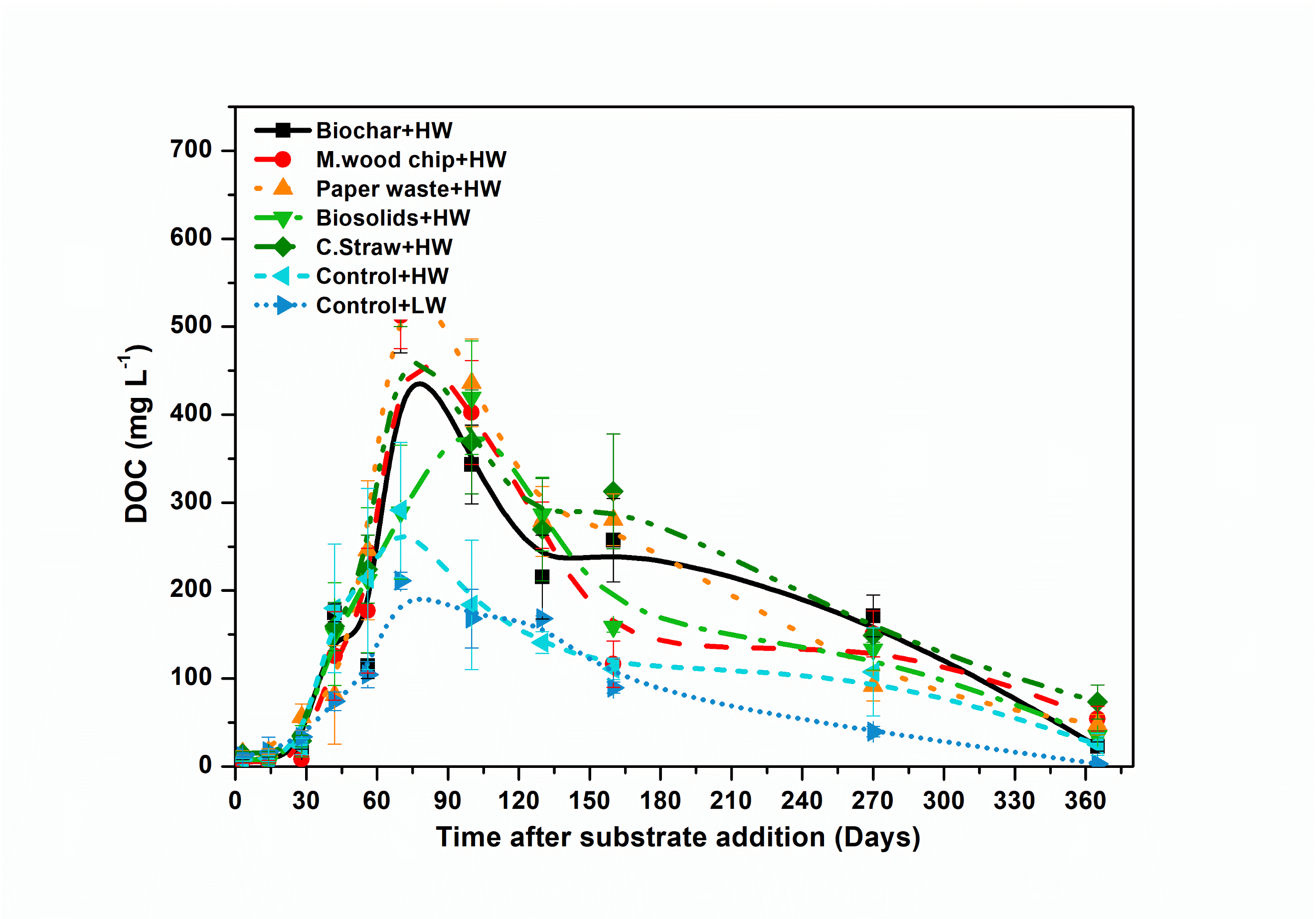

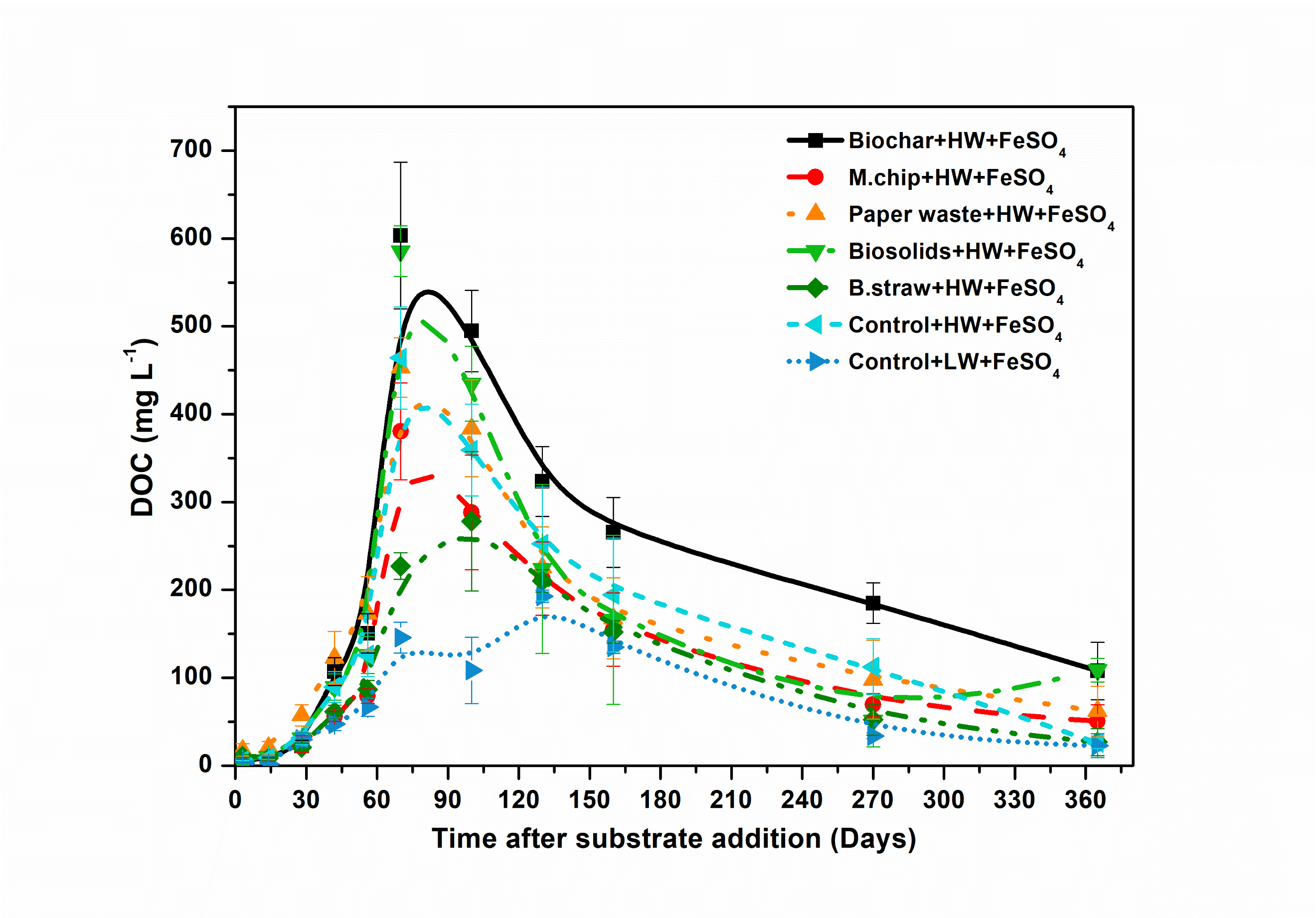


**Fig. S1.** Effect of organic C amendment and FeSO_4_ addition on changes in soil chemical properties in an agricultural peat soil throughout a year. soil DOC (a,b), ammonium concentration (c,d). The C amendments included *Miscanthus* biochar, *Miscanthus* chips, paper waste, biosolids or cereal straw. The experiment had two control consisting of a low water table (LW) treatment and high water table (HW) without C amendement with FeSO_4_ addition. Values represent means ± standard errors (*n* = 4).

**Fig. S2**

Panel (a)


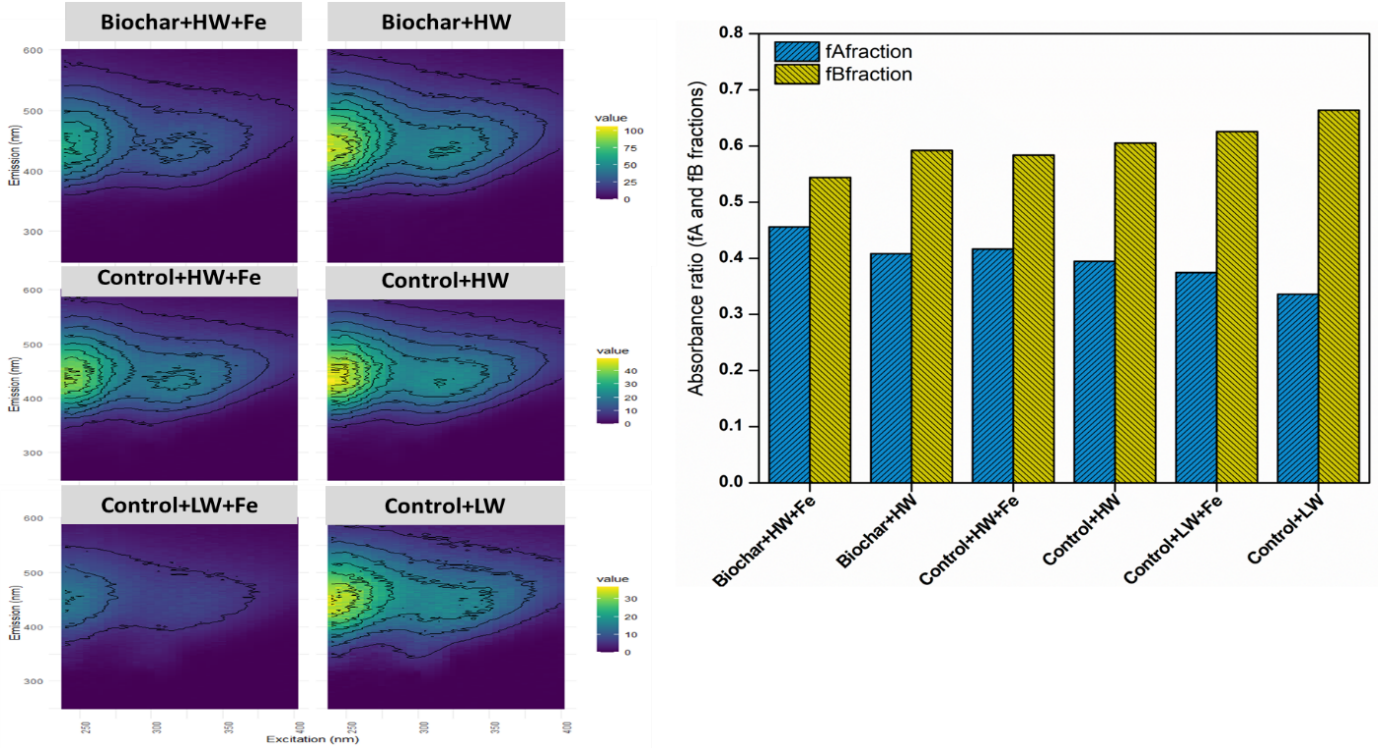


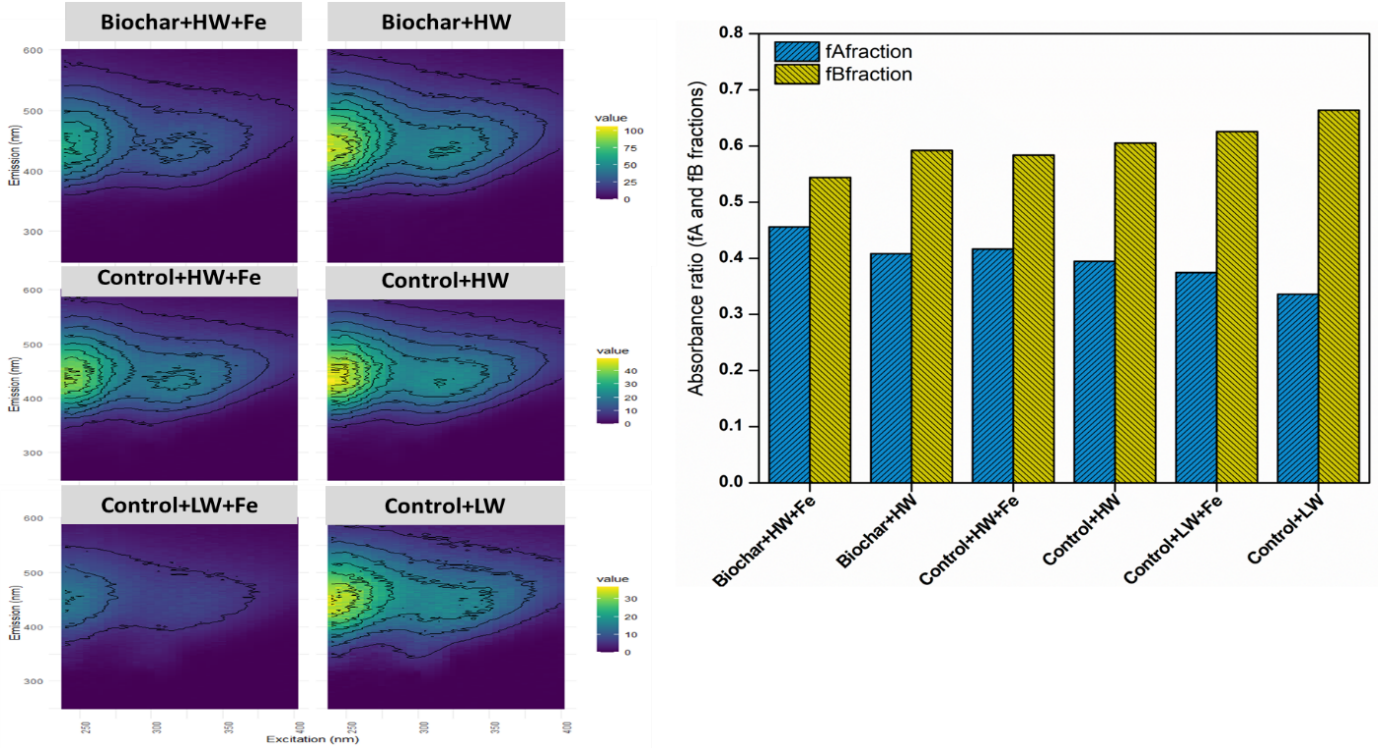


**Fig. S2.** Effect of organic C amendment and FeSO_4_ addition on changes in Panel (a) dissolved organic matter (DOM), Panel (b) specific ultraviolet absorbance at 254 nm of DOC (SUVA_254_), and Panel (c) and absorption ratio of fA and fB fractionsin soil solution from an agricultural peat soil after one year of treatment. The C amendments were loaded at 20 t C ha^-1^ and included biochar (pyrolysed *Miscanthus giganteus* wood chip), commercial paper waste, *M. giganteus* derived chip, barley straw and advanced anaerobically digested biosolids. The experiment had two control consisting of a low water table (LW) treatment and high water table (HW) without C amendement with FeSO_4_ addition ± at a rate of 0.5 t ha^-1^. Values represent means ± standard errors (*n* = 4).

Panel (c)

Panel (b)

(c)

**Fig. S3.**


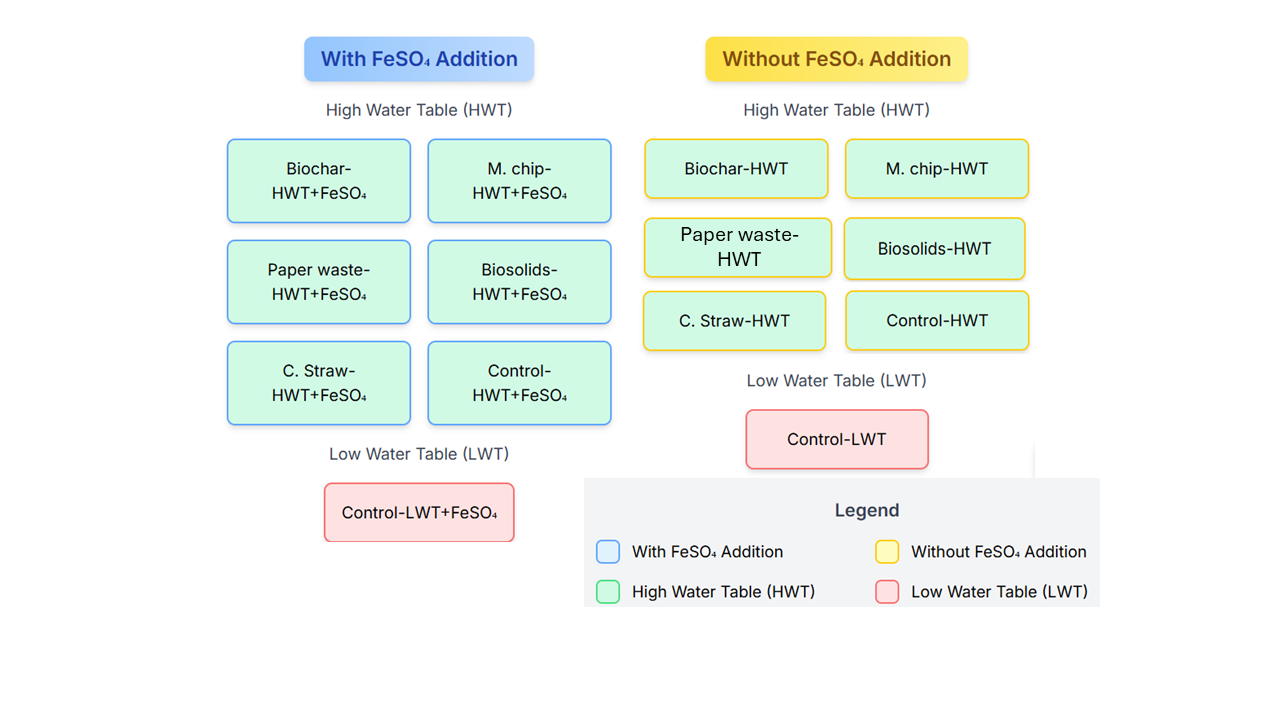

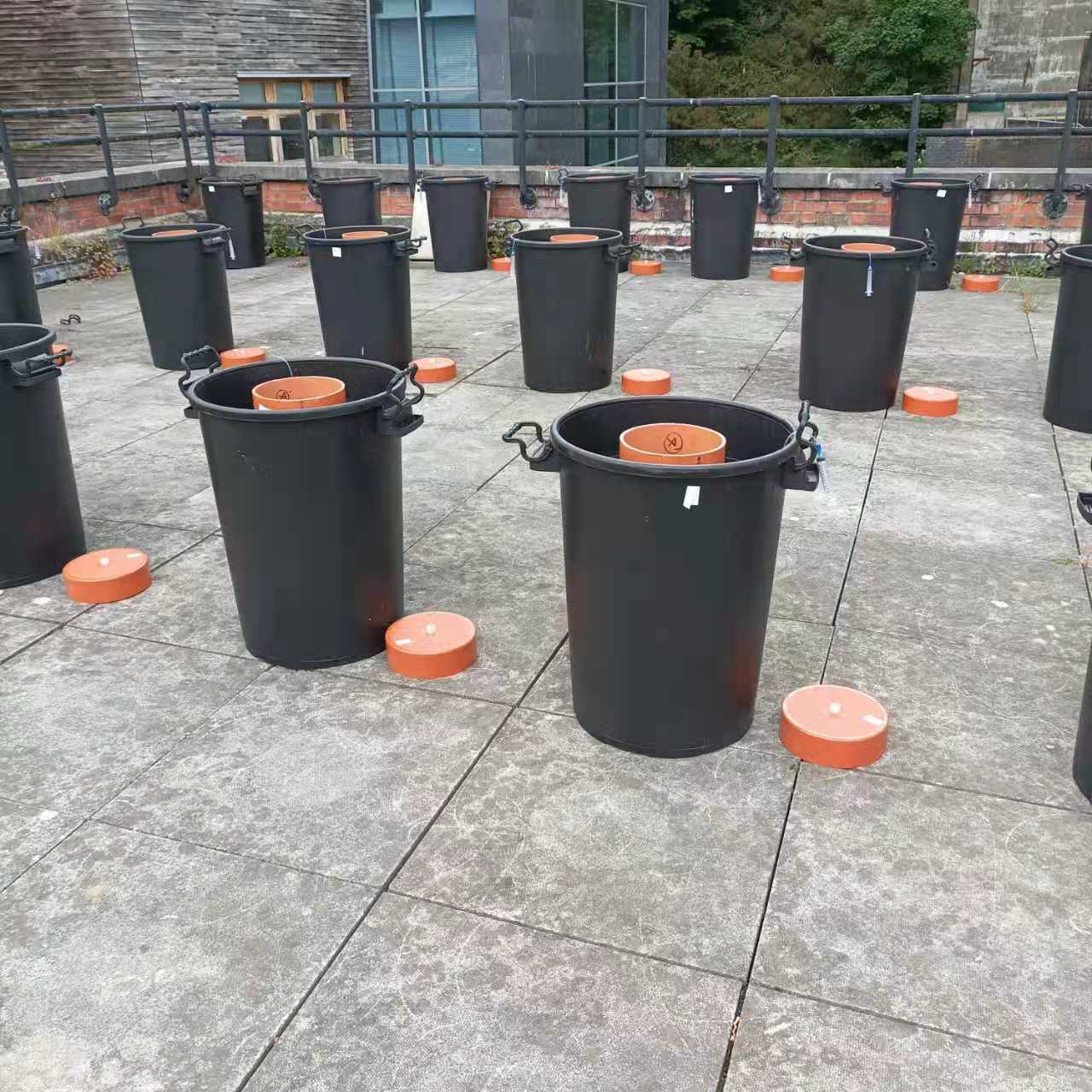


**Panel (A)**

**Panel (B)**

**Fig. S3.** Schematic diagram of the mesocosm experimental set-up (Panel A) and Peat soil mesocosm setup (Panel B). Intact peat soil mesocosm was placed into an outer container with drainage holes drilled to maintain the high or low water table level

Fig. S4


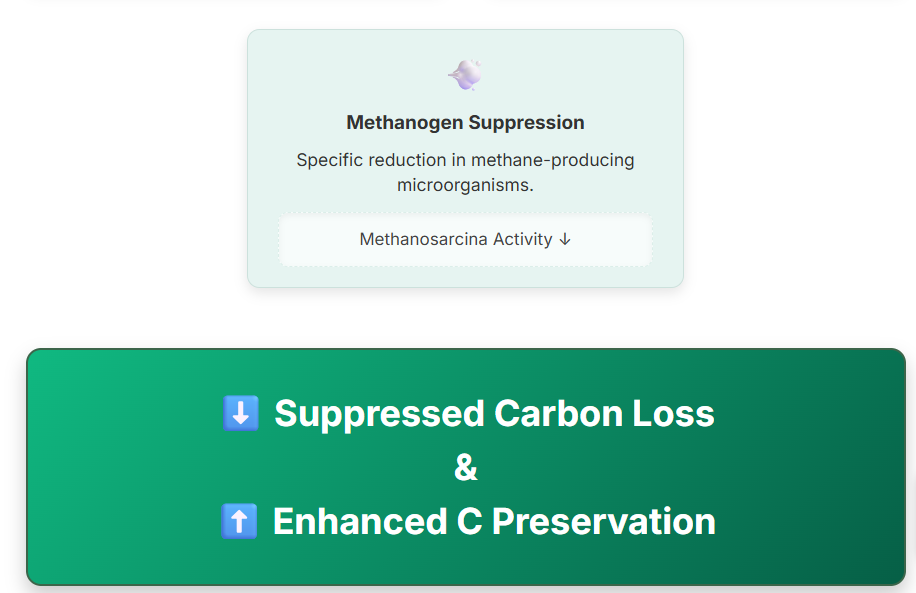

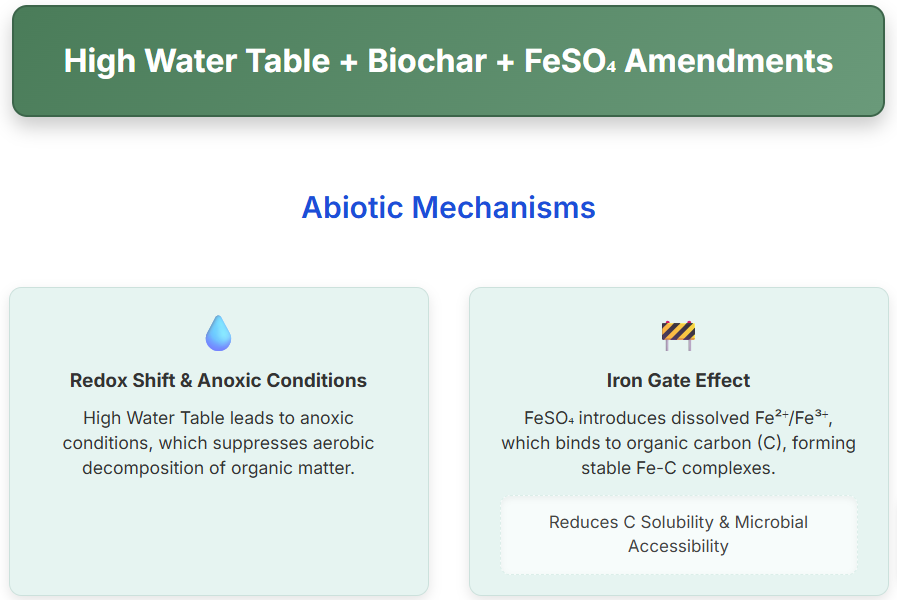

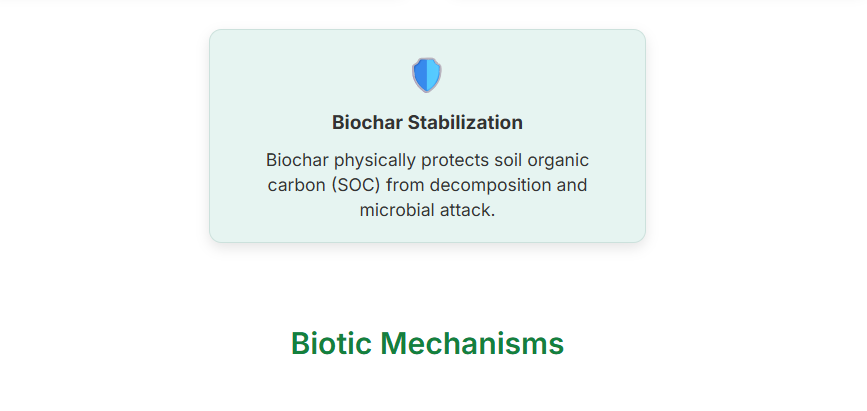

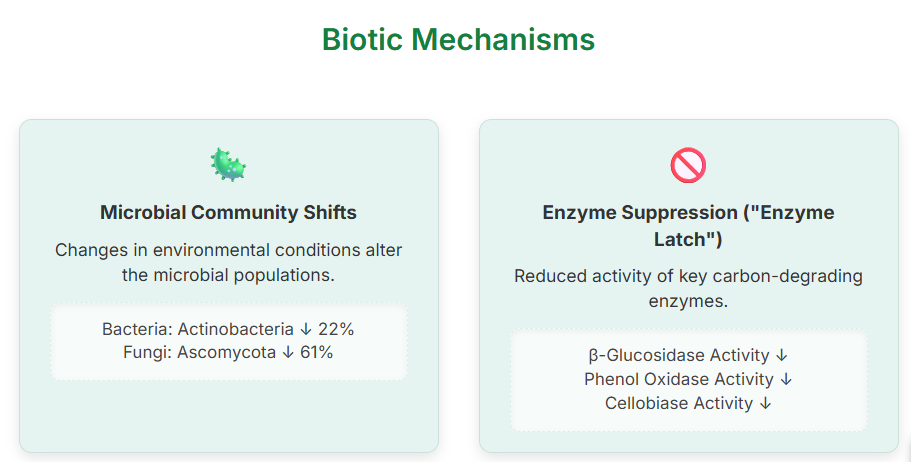

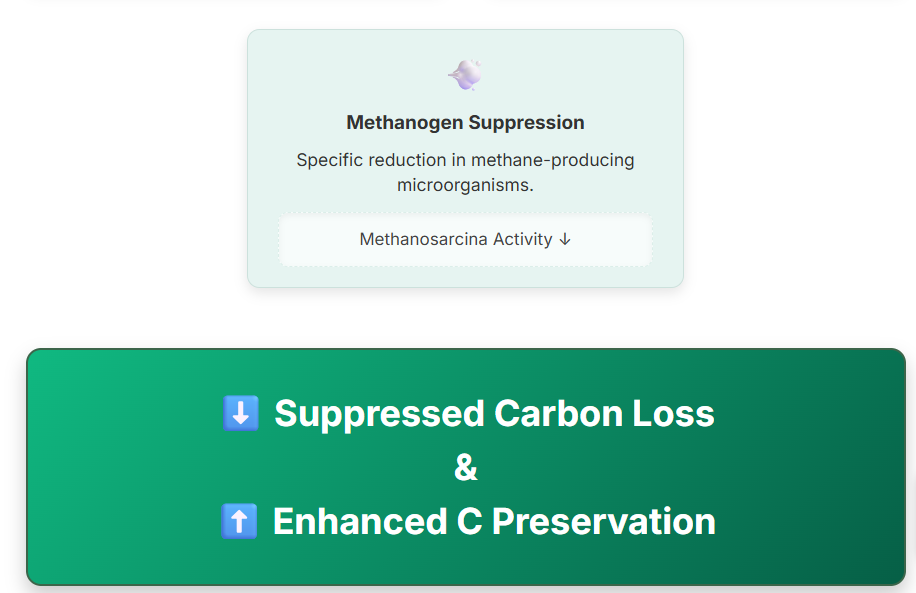


**Fig. S4** Schematic figure that summarizes the abiotic and biotic effects of enzyme latch and iron gate mechanisms with microbial community shifts in two different water table levels and with the addition of biochar and FeSO_4_ soil.

**Table S1.** Physiochemical properties of soil and applied amendments used in the experiments. Values represent means ± standard errors (*n* = 4). Abbreviation: C, carbon; N, nitrogen; OM, organic matter content; EC, electrical conductivity; BD, bulk density. Where applicable, the data is expressed on a dry weight basis. The C amendments were loaded at 20 t C ha^-1^ and included biochar (pyrolysed *Miscanthus giganteus* wood chip), commercial paper waste, *M. giganteus* derived chip, barley straw and advanced anaerobically digested biosolids.

| **Properties** |  | **Soil** |  | **Biochar** | | **M.chip** | | **Paper waste** | | **C.Straw** | **Biosolids** |
| --- | --- | --- | --- | --- | --- | --- | --- | --- | --- | --- | --- |
| Total C (%) | | 27.6±2.6 | | | 79.3±0.9 | | 47.4±0.5 | | 46.8±0.81 | 43.7±0.5 | 36.5±0.3 |
| Total N (%) | | 1.81±0.45 | | | 0.41±0.01 | | 0.49±0.07 | | 0.75±0.07 | 0.68±0.02 | 3.66±0.06 |
| C:N ratio | | 16.0±4.8 | | | 259±14 | | 97.9±16 | | 62.8±7.3 | 63.5±2.0 | 9.95±0.03 |
| OM (%) | | 50.3±1.8 | | | - | | - | | - | - | - |
| pH (H_2_O) | | 6.54±0.05 | | | - | | - | | - | - | - |
| EC (mS cm^−1^) | | 0.2±0.004 | | | - | | - | | - | - | - |
| BD (g cm^-3^) | | 0.52±0.05 | | | - | | - | | - | - | - |
| NO_3_^-^ (mg N L^-1^) | | 4.05±0.29 | | | 0.15±0.01 | | 0.11±0.02 | | 0.3±0.03 | 5.84±0.98 | 1.7 ±0.2 |
| NH_4_^+^ (mg N L^-1^) | | 4.48±0.22 | | | 0.25±0.08 | | 2.87±0.56 | | 1.02±0.08 | 3.79±1.02 | 218±0.15.6 |
| SO_2_^2-^ (mg S L^-1^) | | 1.35±0.42 | | | - | | - | | - | - | - |
| PO_4_^3-^ (mg P L^-1^) | | 1.05±0.11 | | | 3.33±0.98 | | 2.26±0.8 | | 3.57±0.9 | 4.03±0.89 | 2.32±0.23 |

**Table S2. Summary of** the experimental conditions employed in different treatments.

| Treatments | With FeSO_4_ addition | | Without FeSO_4_ | |
| --- | --- | --- | --- | --- |
|  | HW | LW | HW | LW |
| Biochar | Biochar-HW+FeSO_4_ |  | Biochar-HW |  |
| Miscanthus chip | M. chip-HW+FeSO_4_ |  | M. chip-HW |  |
| Paper waste | Paper waste-HW+FeSO_4_ |  | Paper waste-HW |  |
| Biosolids | Biosolids-HW+FeSO_4_ |  | Biosolids-HW |  |
| Cereal straw | C. Straw-HW+FeSO_4_ |  | C. Straw-HW |  |
| Control | Control-HW+FeSO_4_ | Control-LW+FeSO_4_ | Control-HW | Control-LW |

Abbreviations: HW; high water table at -0 cm from the top of soil mesocosm, LW; Low water table at -40 cm from the top of soil mesocosm

**Calculation**

The soil sample was extracted with sodium chloride (NaCl) with an equivalent ionic strength to trisodium citrate and sodium dithionite. The soil residues were rinsed three times with 1 M NaCl and dried. Then OC was used to determine Fe-SOC and the fraction of Fe bound SOC to total SOC in soil (f_Fe-SOC_) were calculated as follows:

Fe-SOC = OC_NaCl_- OC_DCB_  (Eqn. S1)

f_Fe-SOC_ = Fe-SOC / SOC *100% (Eqn. S2)

Iron carbon stoichiometric ratio (Fe/C) = (SOC/12) / (Fe_t_/56) (Eqn. S3)

OC_NaCl_ and OC_DCB_ were the NaCl and DCB-extracted soil residues, respectively.
